# Prevalence of Antibiotic-Resistant Pulmonary Tuberculosis in Bangladesh: A Systematic Review and Meta-Analysis

**DOI:** 10.3390/antibiotics9100710

**Published:** 2020-10-17

**Authors:** Shoumik Kundu, Mahfuza Marzan, Siew Hua Gan, Md Asiful Islam

**Affiliations:** 1 Department of Biochemistry and Molecular Biology, Jahangirnagar University, Savar, Dhaka 1342, Bangladesh; shoumikk33@gmail.com; 2 Department of Microbiology, Jahangirnagar University, Savar, Dhaka 1342, Bangladesh; mmarzan@juniv.edu; 3 School of Pharmacy, Monash University Malaysia, Jalan Lagoon Selatan, Bandar Sunway 47500, Malaysia; gan.siewhua@monash.edu; 4 Department of Haematology, School of Medical Sciences, Universiti Sains Malaysia, Kubang Kerian 16150, Malaysia

**Keywords:** pulmonary tuberculosis, antibiotic resistance, prevalence, epidemiology, Bangladesh, systematic review, meta-analysis

## Abstract

Resistance to anti-tuberculosis (anti-TB) antibiotics is a major public health concern for many high-TB burden countries in Asia, including Bangladesh. Therefore, to represent the overall drug-resistance pattern against TB in Bangladesh, a systematic review and meta-analysis was conducted. Databases such as PubMed, Scopus, and Google Scholar were searched to identify studies related to antibiotic-resistant TB. A total of 24 studies covering 13,336 patients with TB were secured and included. The random-effects model was used to calculate the summary estimates. The pooled prevalence of any, mono, multi, poly, and extensive anti-TB antibiotic-resistances were 45.3% [95% CI: 33.5–57.1], 14.3% [95% CI: 11.4–17.2], 22.2% [95% CI: 18.8–25.7], 7.7% [95% CI: 5.6–9.7], and 0.3% [95% CI: 0.0–1.0], respectively. Among any first and second-line anti-TB drugs, isoniazid (35.0%) and cycloserine (44.6%) resistances were the highest, followed by ethambutol (16.2%) and gatifloxacin (0.2%). Any, multi, and poly drug-resistances were higher in retreatment cases compared to the newly diagnosed cases, although mono drug-resistance tended to be higher in newly diagnosed cases (15.7%) than that in retreatment cases (12.5%). The majority (82.6%) of the included studies were of high quality, with most not exhibiting publication bias. Sensitivity analyses confirmed that all outcomes are robust and reliable. It is concluded that resistance to anti-TB drugs in Bangladesh is rampant and fast growing. Therefore, the implementation of a nationwide surveillance system to detect suspected and drug-resistant TB cases, as well as to ensure a more encompassing treatment management by national TB control program, is highly recommended.

## 1. Introduction

Tuberculosis (TB) is a communicable infectious disease caused by the *Mycobacterium tuberculosis.* Although it mainly affects the lungs (pulmonary TB), it may also involve other parts of the body (extra-pulmonary TB). The World Health Organization (WHO) estimates that approximately one-fourth of the world’s population is infected with *Mycobacterium tuberculosis* which can progress to TB. It has been estimated that 10 million people were infected by TB, with 1.2 million fatalities due to non-human immunodeficiency virus (HIV) TB in 2018. However, TB burden is not uniformly distributed throughout the world. In fact, the top eight countries account for almost 67% cases (86% in 2018 alone) of the total TB patients globally, where Bangladesh ranks 7th in the list and is responsible for 4% of the total worldwide TB cases [1].

Although there are effective treatment regimens consisting of first-line (i.e., streptomycin, isoniazid, rifampicin, ethambutol, and pyrazinamide) and second-line drugs (i.e., fluoroquinolones, amikacin, kanamycin, capreomycin, ethionamide, prothionamide, cycloserine, and para-aminosalicylic acid), successful treatment outcomes are often challenged by the development of drug-resistant (DR) strains, the adverse effects of drugs, and lengthy treatment procedures [2,3,4,5]. The burden of multi-DR-TB (MDR-TB; resistance to both isoniazid and rifampicin) and extensively-DR-TB (XDR-TB; MDR-TB along with resistance to fluoroquinolones and one of the second-line injectable drugs) are expected to increase in countries with high-burden TB. Some of the major underlying risk factors for DRs in patients with TB include treatment failure, relapse, acquired DR, lack of drug susceptibility testing (DST), limited drug supply, and indiscriminate use of anti-TB drugs as well as the cost of DST. Unfortunately, due to the high cost of DST, many newly diagnosed patients are treated with anti-TB drugs without implementing DST in low and middle-income countries [6,7,8].

According to the first nationwide survey conducted in Bangladesh (2010–2011), 18.7% patients with TB showed resistance to any one of the first-line drugs and the rate of MDR-TB was 7.0% (new cases: 1.4% and retreatment cases: 28.5%) [9]. In addition, a sentinel surveillance conducted during 2011–2014 in Bangladesh detected a rise in the resistance to any of the first-line drugs (22.3%) as well as MDR-TB (2.3%) in newly diagnosed cases [10]. Following the initiation of the “directly observed treatment, short-course” (DOTS) program in Bangladesh in 1993, there was a tremendous success in TB treatment. Since 2005, more than 91% of the pulmonary-TB patients have recovered, with more than 95% successfully treated in 2016 [11].

Recent studies in several countries have illustrated that the prevalence of any anti-TB-DR was 20–43% in the total population, with higher prevalence seen in previously treated TB patients. Based on some recent meta-analyses, the burden of MDR-TB was approximately 5 to 7 times higher in retreated patients as opposed to the newly diagnosed patients [12,13,14,15]. Due to the persistent augmentation of MDR-TB cases, resistance to second-line anti-TB drugs are common. In Bangladesh, a short nine-month treatment regimen (known as “Bangladesh regimen”) including gatifloxacin, clofazimine, ethambutol, and pyrazinamide throughout the treatment period supplemented by prothionamide, kanamycin, and high-dose of isoniazid have been confirmed to be highly efficient in treating MDR-TB patients with a success rate of 87.9% [3,16]. Although the treatment of MDR-TB in Bangladesh generally has a success rate of more than 70%, the government presumes that they require more case detections, DST, high-quality laboratories, and instruments for a better prediction on the disease trajectory in the country [1,11].

Consolidated data on DR can illustrate a huge portrait of the present situation to policy makers, physicians, and the scientific community at large, thus enabling a better plan in combating DR patterns and upholding successful treatment outcomes. To date, since no study has previously attempted to gather information on TB-DR patterns in Bangladesh, a systematic review with meta-analysis of published data on DR patterns in patients with pulmonary-TB in Bangladesh is conducted.

## 2. Results

### 2.1. Study Selection

**Figure 1:**
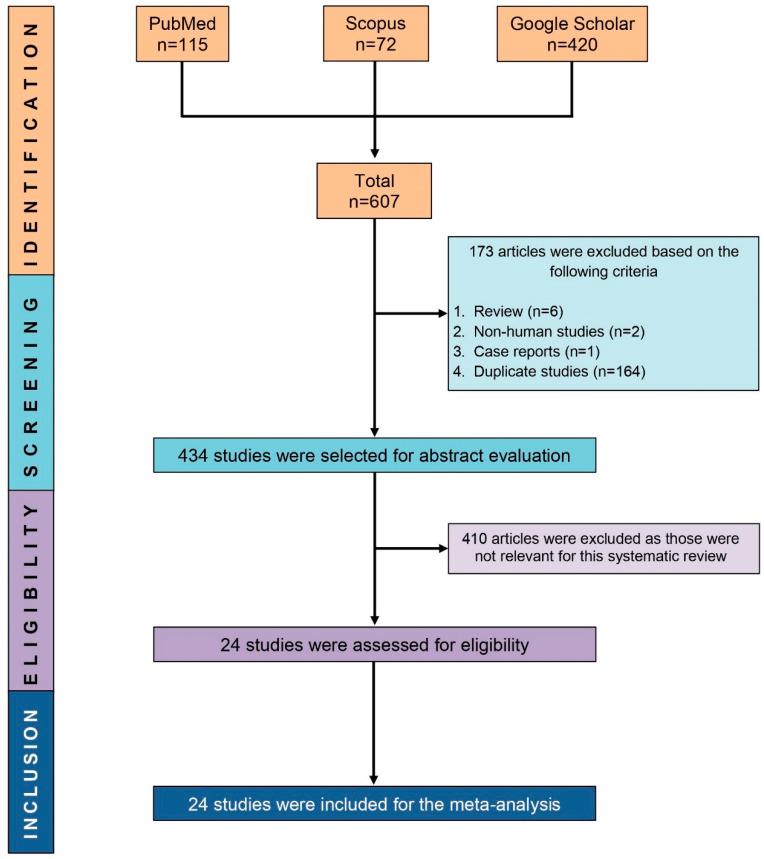
PRISMA flow diagram showing the process of selecting eligible studies.

Based on the search results, 607 studies were identified. After removing 173 studies (164 duplicate studies, six review articles, two non-human studies, and one case report), the abstracts of 434 studies were evaluated for eligibility. Finally, 24 studies were deemed to be eligible and were included in the systematic review and meta-analysis (Figure 1).

### 2.2. Study Characteristics

In this meta-analysis, the data from 13,336 TB patients from Bangladesh (27.5% female) were reported. The ages of the TB patients ranged between 0.0 and 90.0 years. The detailed characteristics and references of the included studies are presented in Table 1.

### 2.3. Quality Assessment

**Figure 2:**
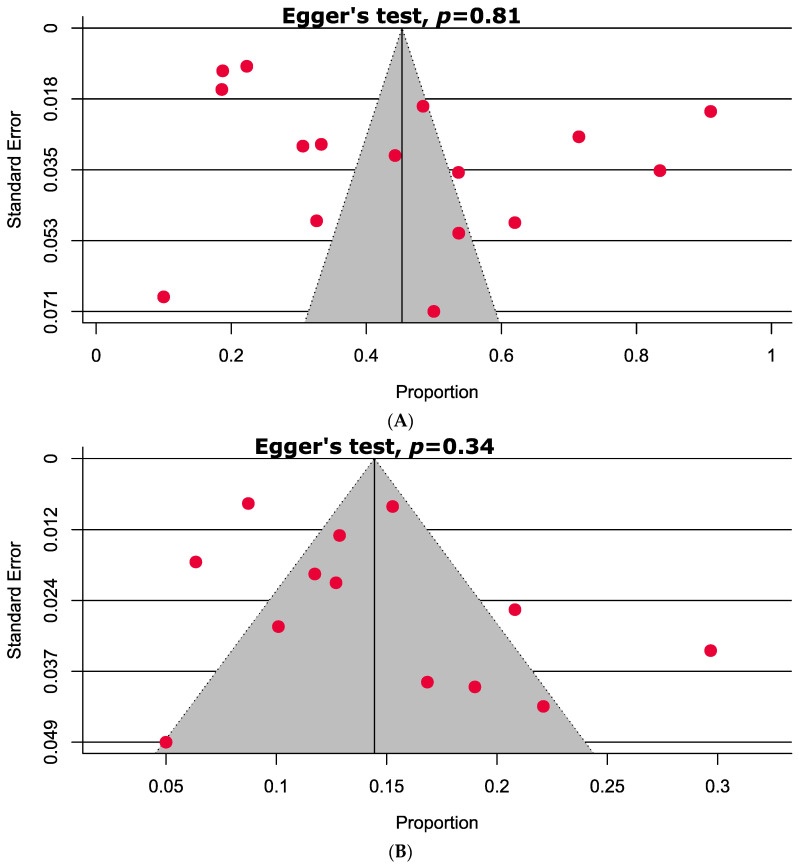

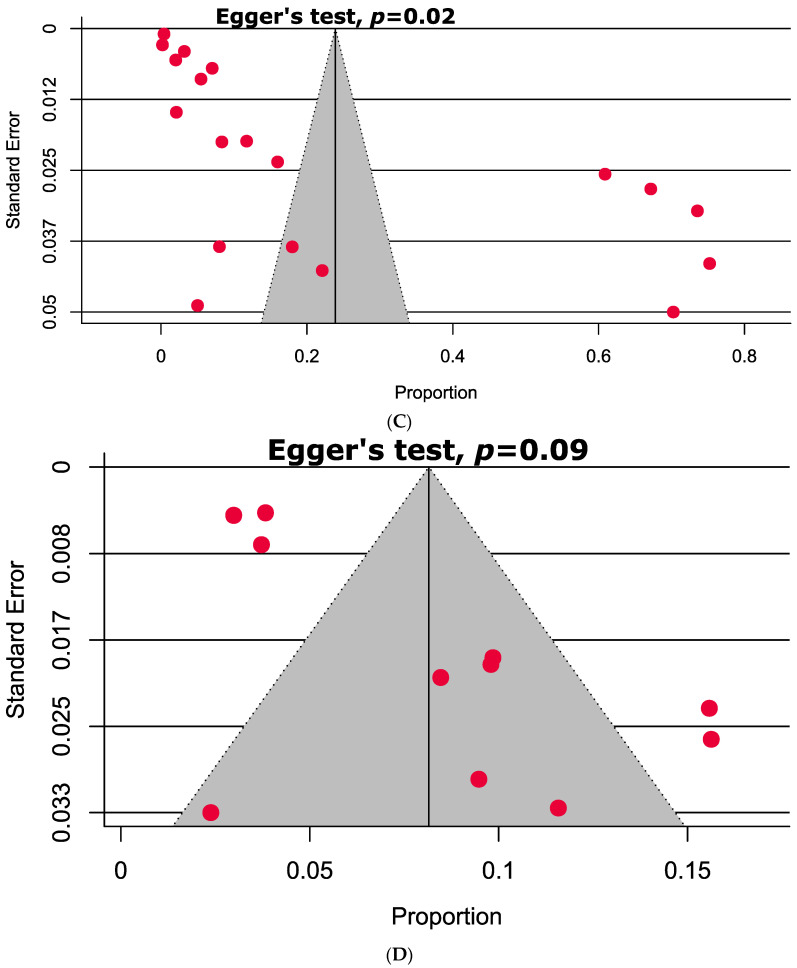
Funnel plots analyzing publication bias among studies evaluated (**A**) any-DR, (**B**) mono-DR, (**C**) multi-DR, and (**D**) poly-DR.

A detailed quality assessment of the included studies is shown in the Supplementary Materials (Tables S2 and S3). Briefly, 82.6% of the included studies were of high quality (with a low risk of bias). A visual inspection of the funnel plot and Egger’s test results indicated that only a single analysis exhibited a significant publication bias (Figure 2).

### 2.4. Outcomes

**Figure 3:**
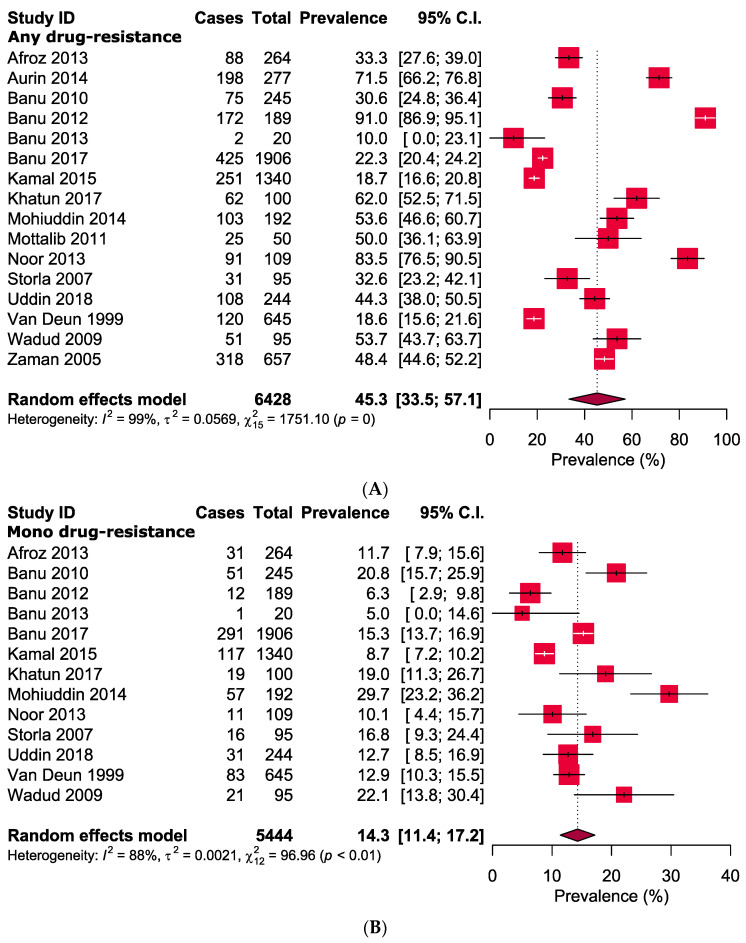

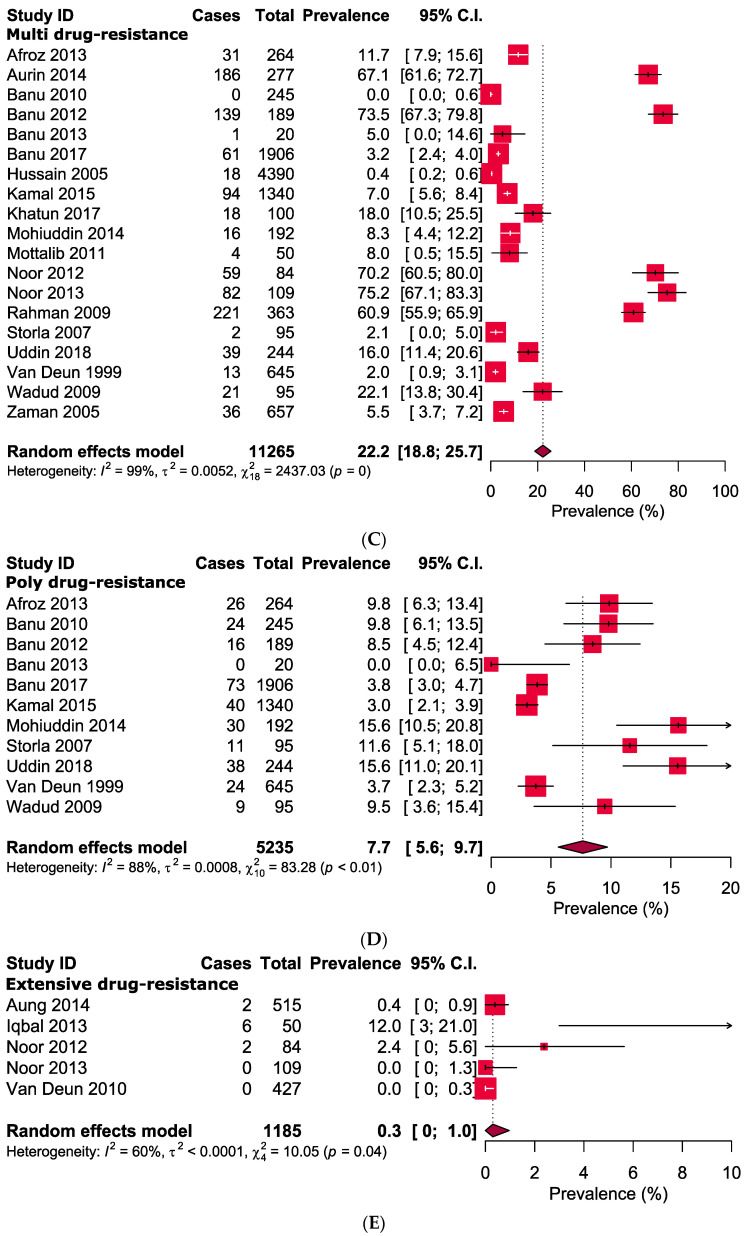
Prevalence of (**A**) any-DR, (**B**) mono-DR, (**C**) multi-DR, (**D**) poly-DR, and (**E**) extensive-DR in pulmonary tuberculosis in Bangladesh.

Overall, the pooled prevalence of any, mono, multi, poly, and extensive anti-TB antibiotic resistances were 45.3% [95% CI: 33.5–57.1], 14.3% [95% CI: 11.4–17.2], 22.2% [95% CI: 18.8–25.7], 7.7% [95% CI: 5.6–9.7], and 0.3% [95% CI: 0.0–1.0], respectively (Figure 3). Among the any-DR, the prevalence for all first-line drugs were high (ranging from 16.2% for ethambutol to 35.0% for isoniazid) (Table 2, Figures S1 and S2). Additionally, variable degrees of resistance patterns were also observed among any second-line drugs, where gatifloxacin showed the lowest (0.2%) and cycloserine exhibited the highest (44.6%) prevalence. (Table 2, Figures S1 and S2).

Among the newly diagnosed TB patients, any resistance to streptomycin (21.4%) and ofloxacin (5.7%) were the highest among the first- and second-lines anti-TB drugs, respectively (Table 3 and Figure S3). On the other hand, among the previously diagnosed patients with TB, three of the first-line anti-TB drugs depicted a resistance prevalence of more than 40% (isoniazid: 49.9%, streptomycin: 42.5%, and rifampicin: 40.9%) while the resistance to ofloxacin (9.4%) was the highest among the second-line drugs. In the mono-DR of first-line drugs, the prevalence ranged from 0.0% (pyrazinamide) to 6.1% (streptomycin).

**Figure 4:**
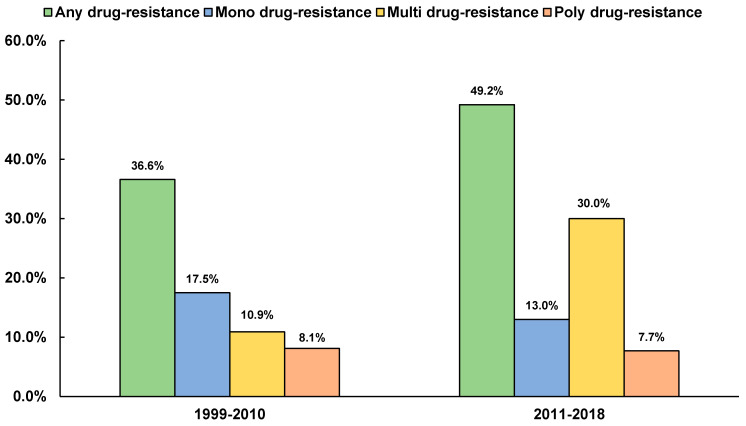
Anti-tuberculosis antibiotic resistance patterns in Bangladesh between 1999 and 2018.

The majority of the newly (9.3%) and previously diagnosed (6.5%) mono first-line-DR-TB patients were resistant to streptomycin (Table 3 and Figure S4). The prevalence of MDR-TB was substantially higher in the previously treated cases (20.9%) than in the newly diagnosed cases (1.3%). On the other hand, the prevalence of poly-DR was two-folds in retreatment cases (8.6%) as compared to that in the new cases (4.0%) (Figure S5). Additionally, an interesting trend of increasing resistance pattern was observed in any (from 36.6% to 49.2%) and multi-DR (from 10.9% to 30.0%). On the other hand, poly-DR was found to be almost stable in the last 20 years while the mono-DR followed a decreasing trend (i.e., from 17.5% to 13.0%) (Figure 4).

### 2.5. Sensitivity Analyses

Our sensitivity analyses, which excluded small and low-quality studies, showed marginal differences in the overall pooled prevalence compared to the main findings with the exception of the analysis with studies on extensive-DR (Table 4 and Figure S6). Overall, our sensitivity analyses indicated that all the outcomes are robust and reliable.

## 3. Discussion

To the best to our knowledge, this is the first study to report on the prevalence of DR-TB in Bangladesh where there is a high prevalence of any anti-TB-DR in the overall study population (45.3%). In comparison to several recent meta-analyses conducted in different countries, the proportion of any-DR in both newly diagnosed and previously treated cases is significantly higher in Bangladesh. Nevertheless, compared to Bangladesh, a study in Iran demonstrated a higher prevalence of DR in retreatment cases (65.6%) and another study in Nigeria showed a higher rate of DR in new cases (32.0%). Moreover, the prevalence of any anti-TB-DR in the overall population was also higher in Bangladesh than that of India (42.6%) [12,13,14,15].

### 3.1. Prevalence of Any and Mono-Resistance to First-Line Anti-TB Drugs

Among the first-line anti-TB drugs, any resistance to isoniazid was the highest in the overall study population (35.0%) and in retreatment cases (49.9%) in Bangladesh. In fact, the prevalence of any isoniazid-resistance in retreatment patients was higher than that reported in Iran (47.0%) or in Pakistan (37.1%). However, a study in Hangzhou and another study in Shanghai, two cities in China, illustrated different prevalence of any isoniazid-resistance in retreatment cases: 29.0% and 53.2%, respectively. On the other hand any resistance to isoniazid in new patients (10.8%) was comparatively lower in Bangladesh than that in India, China, and Iran [13,35,36,37,38]. Any streptomycin-resistance was substantially high in Bangladesh among new (21.4%), retreatment (42.5%) and overall study subjects (29.2%). Streptomycin resistance rate in retreatment cases in Bangladesh is somehow comparable to the resistance rate found in Iran (44.2%) and in India (43.3%), although Bangladesh showed a higher streptomycin resistance rate among new patients than other countries. Any rifampicin-resistance in retreatment patients was also markedly higher in Bangladesh (40.9%) than that reported in Pakistan (25.2%) and Iran (38.2%) but was lower than that of China (48.7%) and India (54.3%). Any ethambutol-resistance in retreatment patients was between 24% and 31% in several countries as compared to 27.6% in Bangladesh [13,35,36,39].

The rate of mono-resistance in the overall Bangladeshi population was high (14.3%) compared to that in Nigeria and China but was lower when compared to that in Iran (17.1%) [13,14,15]. Mono-resistance to all first-line anti-TB drugs was higher in the overall population in India as compared to that in Bangladesh [12]. Among the FLDs mono resistance, the highest resistance was observed against streptomycin in the overall population (6.1%) as well as in new (9.3%) and retreatment cases (6.5%). Streptomycin mono resistance was higher in Bangladesh compared to the rate found in South Africa [40] and China [37]. However, the mono resistance to streptomycin in new cases was higher in Iran (13.2%) than that observed in Bangladesh (9.3%) [41]. The mono resistance to rifampicin (0.7%) and ethambutol (0.8%) was lower than that reported for the recent studies in India [12], Peru [42], and South Africa [40].

### 3.2. The Molecular Basis and Influential Factors for the Resistance to First-Line Anti-TB Drugs

The molecular basis of DR in TB is the result of the presence of mutations in the first-line anti-TB drug-target genes or in the genes that encode essential enzymes for those drug activation [43]. For instance, mutations in *katG* and *inhA* genes were significantly associated with isoniazid resistance and approximately 95% of the rifampicin-resistant TB patients carry mutations in the *rpoB* gene [44]. Mono resistance to rifampicin is uncommon and in most cases rifampicin resistant strains are also resistant to isoniazid. Rifampicin resistance, therefore, acts as a representative marker of MDR-TB [43,45]. A low level of rifampicin resistance may be attributed to the less availability of it in the private pharmacies as most people rely on those private pharmacies for purchasing their medications [9]. Use of streptomycin for TB treatment is tremendously decreased in developed countries because of its parenteral administration procedure and adverse effects; however, in Bangladesh it is still used for retreatment cases. Therefore, the high resistance to streptomycin, which may be caused by its application in non-TB diseases as well, may affect retreatment case management adversely [9,45].

### 3.3. Prevalence of MDR-TB and Its Influential Factors

The prevalence of MDR-TB in both new (1.3%) and retreatment cases (20.9%) in Bangladesh was lower than that of India [12], Iran [13], Nigeria [14], China [15,35], and Pakistan [36,46]. However, the overall prevalence of MDR-TB (22.2%) is higher in Bangladesh than the rate of MDR-TB in above mentioned countries, except India. The Global Tuberculosis Report by WHO [1] illustrated that 1.5% of the new and 4.9% of the previously treated TB cases had MDR/rifampicin resistant TB in Bangladesh which is lower than that of neighboring countries like India, Myanmar, and Pakistan.

There is no single genetic change that directly contributes to the development of MDR phenotype rather improper treatment that causes the sequential mutation at different loci creating a MDR strain [43,47]. A study exploring the risk factors of MDR-TB in Bangladesh found that younger age (<40 years), previous contact, and disease history substantially bolster the risk of getting MDR-TB [48]. According to a study on Chinese population [49], the male sex was identified as a risk factor for developing MDR-TB, however, it was not regarded as an influential factor in Bangladesh [48].

### 3.4. Prevalence of Poly-DR and XDR-TB and Its Influential Factors

The proportion of XDR-TB (0.3%) is lower in Bangladesh compared to that of India (1.9%) [12] and China (2.0%) [50] and quite similar to that of Pakistan (0.4%) [36]. The poly-DR was 7.7% in the overall population; 8.6% in the retreatment patients and 4.0% in the newly diagnosed patients in Bangladesh. A study conducted in Sudan [51] showed poly-DR rate of 6.4% among the retreatment cases while in another surveillance study in Canada [52], the poly-DR was only 0.4%. Indiscriminate use and self-medication of fluoroquinolones were previously reported in Bangladesh which may act as an underlying reason for XDR-TB [9].

### 3.5. Prevalence of Second-Line Anti-TB-DR and Its Associated Factors

Among the second-line anti-TB drugs, the resistance pattern of kanamycin, ofloxacin, and gatifloxacin was well-investigated in Bangladesh (analyzed in at least four original studies). Among the different drugs, gatifloxacin and kanamycin were highly efficacious in TB patients, showing a resistance rate of only 0.2% and 0.5%, respectively. However, resistance to ofloxacin was high in overall population (7.3%) and in retreatment cases (9.4%). Among these drugs, kanamycin and ofloxacin resistance were significantly higher in China and Europe as compared to that in Bangladesh. In another study based in Pakistan, ofloxacin resistance was approximately three times higher (27.4%) in the previously treated cases although resistance to kanamycin was reported to be slightly lower (0.4%). Moreover, a high prevalence of kanamycin (6.5%) and ofloxacin (9.7%) resistance was reported in India [35,36,39,53]. A recent study illustrated that the fluoroquinolone resistance in MDR-TB patients was correlated with first-line anti-TB-DR and the genetic mutations in *gyrA* and *gyrB* genes [54]. However, the lower rate of resistance to gatifloxacin is beneficial for Bangladesh as this is a core drug used in the short regimen for MDR-TB [4]. Moreover, gatifloxacin is more impervious to microbial resistant than ofloxacin because of its structure [55].

To date, resistance to other second-line anti-TB drugs was analyzed in a very small number of studies in Bangladesh. However, among the drugs, cycloserine resistance was reported to be the highest (44.6%). Resistance to ethionamide (35.1%), prothionamide (15.7%), and para-aminosalicylic acid (12.2%) was also substantially high in Bangladesh. In fact, resistance to para-aminosalicylic acid was slightly higher in a study conducted on MDR-TB patients in Thailand (14.14%). Resistance to amikacin was only 1.4% in Bangladesh. However, the prevalence was higher (2 to 5%) in different studies from China [35], the Netherlands [56], and Thailand [57] and is lower (0.5%) in a study from Pakistan [36]. In the Netherlands, resistance to prothionamide (8%) and cycloserine (1%) was significantly lower than that in Bangladesh. [56]. However, in the studies of Canada and Netherlands a major portion of the DR-TB patients were the foreign migrants and the improper use of SLDs were presumed to be the principal cause of their DR [52,56].

### 3.6. Time-Trend of Anti-TB-DR in Bangladesh Compared to Other Countries

Our 20-year analysis depicted that the number of MDR-TB patients in Bangladesh increased approximately 1.76 times when the data was compared between 1999–2010 and that for 2011–2018. Other studies in different countries also illustrated a similar increase in MDR-TB cases with time. In India, the prevalence of MDR-TB was augmented by 1.87 times when the data was compared between 1995 and 2005 to between 2006 and 2015. In China and South Africa, the increase was approximately 1.81 times from 2006 to 2012 (for China) and 1.31 times from 2001–2002 to 2012–2014 for South Africa. Additionally, China reported a 19.5% increase in any-DR from 2006 to 2012, as compared to 12.6% for Bangladesh between 1999–2010 and 2011–2018. Similar to Bangladesh, India showed a decreasing trend in mono resistance [12,40,58].

### 3.7. Recommendations to Prevent Further Acceleration of Anti-TB-DR in Bangladesh

In order to prevent the further acceleration of TB-DR, several factors which are influential for the development of DR-TB and MDR-TB in Bangladesh should be given immediate attention. A strong implementation and regular review of the DOTS program to ensure a strict adherence to treatment is highly recommended, especially in semi-urban and rural areas where the risk of developing MDR-TB is 3 and 14 times higher, respectively, as compared to urban areas [48,59]. In fact, attrition rates of 17% for pre-diagnostic (suspected cases not tested) and 21% for pre-treatment (delay between diagnosis and treatment initiation) have previously been reported and need to be addressed [11,60].

The NTP in Bangladesh requires the incorporation of regular surveillance of DR patterns which is an indispensable component of a sustainable TB control program and can act as an epidemiological indicator to its success. Steps such as the augmentation of national budget, case detection in remote areas, HIV detection and laboratory establishment should be prioritized. The regular screening of high-risk groups such as on individuals living below the poverty line, people with risk factors (smoking, HIV, and type 2 diabetes), those in contact with TB patients is of utmost importance for early case detection and to decline the spread of DR-TB.

Monitoring treatment delay following detection is also required to avoid serious consequences. DST should be made compulsory for retreatment patients since they have a higher possibility of developing DR-TB. In addition, the number of studies that assessed the resistance pattern of second-line anti-TB drugs remain very low in Bangladesh to predict the actual scenario of the prevalence of resistance of TB patients to the drugs. Therefore, adopting necessary policies for increasing case detection, DST, economic stability, and laboratory facilities are strongly recommended to combat DR-TB in Bangladesh.

### 3.8. Strength and Limitations

Our study has several strengths. The meta-analysis is the first, to our knowledge, to comprehensively investigate the prevalence of anti-TB antibiotic-resistance in TB patients in Bangladesh. This meta-analysis was conducted with a significant number of studies and therefore included a considerable number of participants, resulting in more robust estimates. None of the analyses represented significant publication bias, demonstrating that we were unlikely to have missed studies that could have altered the findings. All the conducted sensitivity analyses generated very similar results to the main findings indicating the robustness of the meta-analysis. Additionally, most of our included studies were of high methodological quality (low risk of bias), which ensured a reliable result. Nevertheless, as with other studies, there were some limitations. Even though publication bias was not obvious from none of the analyses, the Egger’s test has limitation in detecting publication bias in case of smaller number of studies. Most of the analyses generated substantial degrees of heterogeneity. Although the sources of heterogeneity by subgroup and sensitivity analyses have been examined, they could not be fully explained by the factors included in the analyses. Further studies should explore the resistance pattern of second-line anti-TB drugs, molecular characterization of drug resistant strains, and proportion of DR-TB in younger population extensively as they exhibited a higher possibility to have MDR-TB in Bangladesh.

## 4. Materials and Methods

### 4.1. Systematic Review Protocol

The systematic review with meta-analysis was conducted in accordance with the PRISMA guideline [61]. The protocol of the study was registered with PROSPERO (CRD42020206076).

### 4.2. Eligibility Criteria

Studies which reported on the prevalence of antibiotic-resistance in TB patients from Bangladesh were included with no age or sex restrictions. Studies were excluded if they were: (1) conducted in patients with extra-pulmonary TB and (2) have considered TB patients co-infected with HIV infection. No restrictions were applied on the language, year, or type of study design. Nevertheless, review articles, theses, commentaries, case reports, opinions, and meeting abstracts were excluded.

### 4.3. Search Strategy

A search strategy with relevant keywords including the names of the drugs used for TB treatment, the locations in Bangladesh as well as drug susceptibility and resistance were developed. Searches were then made in PubMed, Scopus, and Google Scholar databases (Table S1). Prior to the abstract evaluation, the references of the studies from the various databases were incorporated into EndNote X8 software. Any duplicated studies were removed.

### 4.4. Study Selection

After removing the duplicated studies, the remaining papers were finally selected for abstract evaluation. The studies were independently screened by three authors (SK, MM, and MAI) where the titles, abstracts and subsequent full texts of the studies were screened for eligibility. Any discrepancies on study inclusion were resolved through discussions among the authors.

### 4.5. Definitions and Data extraction

Any drug resistance (DR): resistance to any antibiotics against TB. First-line drugs: streptomycin, rifampicin, isoniazid, ethambutol, and pyrazinamide. Second-line drugs: kanamycin, ofloxacin, amikacin, ethionamide, prothionamide, moxifloxacin, gatifloxacin, levofloxacin, para-aminosalicylic acid, and cycloserine. Mono DR: resistance to only a single first-line or second-line drugs. MDR: resistance to at least two drugs like isoniazid and rifampicin. Poly-DR: resistance to any drug combination without isoniazid and rifampicin. XDR: An MDR-TB which is also resistant to any of the fluoroquinolones and at least any of the three injectable second-line drugs (i.e., amikacin, kanamycin or capreomycin). Newly diagnosed patients: patients who have never received any drugs against TB. Previously treated patients/retreatment cases: patients who have received anti-TB drugs before for at least one month, patients from this category are: (1) those with treatment failure and/or relapse and (2) those who returned after loss to follow-up.

Data were independently extracted from each of the eligible studies by two authors (SK and MM) and any inconsistencies were discussed with a third author (MAI). Subsequently, the following information were collected and arranged into a spreadsheet: First author’s last name, year of publication, study design, patient enrollment duration, number of patients, antibiotic susceptibility testing method/medium, age of the participants, name of the drugs to which resistance was observed as well as the prevalence of DR.

### 4.6. Quality Assessment

The quality assessment of the eligible studies was done independently by two authors (SK and MM) based on the Joanna Briggs Institute (JBI) critical appraisal tools. Any discrepancies were further resolved through discussions among the authors. The studies were classified as low-quality (high-risk of bias) if the overall score was ≤50%. To assess the publication bias, a funnel plot presenting the prevalence estimate against the sampling variance was constructed and the asymmetry of the funnel plot was confirmed with the Egger’s test, where *p* < 0.05 was considered as statistically significant.

### 4.7. Data Analyses

The random-effects model was used to calculate the pooled prevalence with 95% confidence intervals (CIs). *I*^2^ statistic and Cochran’s Q tests were used to calculate heterogeneity where *I*^2^ value of more than 75% indicated substantial heterogeneity following which *p* < 0.10 was considered as statistically significant. All analyses and plots were generated by using metaprop codes in meta (version 4.11-0) and metafor (version 2.4-0) packages of R (version 3.6.3) in RStudio (version 1.2.5033) [62].

### 4.8. Subgroup and Sensitivity Analyses

In the subgroup analysis, the prevalence of antibiotic resistance in (i) specific first-line and second-line drugs and (ii) resistance patterns in newly diagnosed and previously diagnosed cases were calculated. To identify the source of heterogeneity and to confirm the robustness of the results, sensitivity analyses were performed based on the following strategies by excluding (1) small (*n* < 100) and (2) low-quality studies (high-risk of bias).

## 5. Conclusions

This systematic review and meta-analysis indicated a high prevalence of DR-TB in Bangladesh. Any-DR and multi-DR showed an increasing trend as time progresses. Therefore, a robust national surveillance system equipped with facilities for the rapid detection of TB-DR is highly recommended. Inclusion of all diagnosed patients under effective treatment and monitoring for treatment adherence seems crucial as the DR rate was higher in previously treated patients. It is recommended that the incorporation of a substantial budget, competent workforce, mass social awareness, and up-to-date scientific information be done to assist the NTP in Bangladesh for more viable roles to halt the progression of further antibiotic-resistance to TB.

## Figures and Tables

**Table 1:** Major characteristics of the included studies.

Study ID [References]	Study Design	Location	Patient Enrollment Time	Number of TB Patients Tested for Antibiotic Resistance (Female)	Antibiotic Susceptibility Testing Method/Medium	Age in Years (Mean ± SD/Range)	Anti-TB-DR Evaluated
Afroz 2013 [17]	Cohort	Narsingdi	February 2011 to November 2011	264 (89)	Conventional DST	0.0–≥64.0	SM, INH, RMP, and EMB
Aung 2014 [4]	Cohort	NR	March 2005 to June 2011	515 (151)	Proportion method/Middlebrook 7H11 agar	12.0–76.0	KM, PTN, and PZ
Aurin 2014 [18]	Cross-sectional	Dhaka	May 2012 to April 2013	277 (NR)	LPA	NR	RMP and INH
Banu 2010 [19]	Cross-sectional	Dhaka	October 2005 to September 2007	245 (4)	Proportion method/LJ	10.0–≥40.0	SM, INH, RMP, and EMB
Banu 2012 [20]	Cross-sectional	Dhaka	February 2002 to September 2005	189 (47)	Proportion method/LJ	12.0–70.0	SM, INH, RMP, and EMB
Banu 2013 [21]	Cross-sectional	Dhaka	August 2009 to December 2010	20 (NR)	Proportion method/LJ	0.0–≥65.0	SM, INH, RMP, and EMB
Banu 2017 [10]	Cross-sectional	Nationwide	August 2011 to December 2014	1906 (548)	Proportion method/LJ	8.0–90.0	SM, INH, RMP, and EMB
Heysell 2015 [3]	Cohort	Nationwide	2011 to 2013	74 (23)	MIC/MYCOTB plate	35.0 ± 15.0	KM, OFX, MFX, ETN, AMK, PAS, and CS
Hussain 2005 [22]	Cross-sectional	Rajshahi	January 1998 to December 2004	4390 (NR)	NR	NR	SM, INH, RMP, and EMB
Iqbal 2013 [23]	Cross-sectional	Dhaka	NR	100 (NR)	Slide DST/SLM	NR	SM, INH, RMP, EMB, GFX, KM, and OFX
Kamal 2015 [9]	Cross-sectional	Nationwide	December 2010 to November 2011	1340 (NR)	Proportion method/LJ	≤14.0–≥65.0	SM, INH, RMP, and EMB
Khatun 2017 [2]	Cross-sectional	Chattogram	July 2012 to July 2013	100 (22)	Gene Xpert MTB/RIF (RMP) and conventional DST (LJ)	15.0–76.0	SM, INH, RMP, and PZ
Mohiuddin 2014 [24]	Cross-sectional	Dhaka	April 2005 to September 2010	192 (NR)	Proportion method/LJ	NR	SM, INH, RMP, and EMB
Mottalib 2011 [25]	Cross-sectional	Dhaka	October 2008 to November 2009	50 (NR)	Proportion method/LJ	14.0–70.0	SM, INH, RMP, and EMB
Noor 2012 [26]	Cross-sectional	Dhaka	January 2011 to April 2012	84 (NR)	Proportion method/LJ	NR	OFX, GFX, and KM
Noor 2013 [27]	Cross-sectional	Dhaka	April 2011 to April 2012	109 (NR)	Conventional DST/LJ	NR	INH, RMP, OFX, GFX, and KM
Rahman 2009 [28]	Cross-sectional	Dhaka	January 2008 to December 2008	363 (76)	Proportion method/LJ	≥10.0	SM, INH, RMP and EMB
Storla 2007 [29]	Cross-sectional	Sunamganj	November 2003 to December 2004	95 (NR)	Bactec MGIT 960 System	NR	SM, INH, RMP, and EMB
Uddin 2018 [30]	Cross-sectional	Mymensingh and Netrokona	2004	244 (90)	Proportion method/LJ	11.0–90.0	SM, INH, RMP, and EMB
Van Deun 2010 [16]	Cohort	NR	May 1997 to December 2007	427 (109)	NR	33.8	OFX, KM, and PTN
Van Deun 1999 [31]	Cross-sectional	Mymensingh	December 1994 to June 1995	645 (NR)	Proportion method/LJ	0.0–≥64.0	SM, INH, RMP, and EMB
Wadud 2009 [32]	Cross-sectional	Dhaka	February 2003 to January 2004	95 (NR)	Proportion method/LJ	6.0–68.0	SM, INH, RMP, EMB, and PZ
Zaman 2005 [33]	Cross-sectional	Dhaka and Matlab	June 2001 to June 2003	657 (214)	Proportion method/LJ	15.0–≥45.0	SM, INH, RMP, and EMB
Zignol 2016 [34]	Cross-sectional	NR	2011	955 (NR)	Proportion method/LJ and MGIT 960 System	NR	PZ, OFX, LFX, MFX, and GFX

DST: Drug Susceptibility Test, LJ: Lowenstein-Jensen Media, LPA: Line Probe Assay, MIC: Minimum Inhibitory Concentration, SLM: Sula Liquid Media, SM: Streptomycin, INH: Isoniazid, RMP: Rifampicin, EMB: Ethambutol, KM: Kanamycin, PTN: Prothionamide, PZ: Pyrazinamide, OFX: Ofloxacin, MFX: Moxifloxacin, ETN: Ethionamide, AMK: Amikacin, PAS: Para-aminosalicylic Acid, CS: Cycloserine, GFX: Gatifloxacin, LFX: Levofloxacin, SD: Standard Deviation, NR: Not reported.

**Table 2:** Any and mono anti-tuberculosis-DR patterns in Bangladesh.

Drug-Resistance Patterns		Antibiotics	Number of Analyzed Studies	Total Number of Tuberculosis Patients	Prevalence of Antibiotic Resistance [95% CIs] (%)	Heterogeneity		Publication Bias, Egger’s Test (*p*-Value)
						*I* ^2^	*p*-Value	
**Any-DR**	**First line drugs**	Streptomycin	10	4990	29.2 [17.8–40.6]	99.0%	<0.0001	0.88
		Isoniazid	13	6018	35.0 [23.1–46.8]	99.0%	<0.0001	0.57
		Rifampicin	13	6018	27.6 [19.9–35.4]	99.0%	<0.0001	0.12
		Ethambutol	10	4990	16.2 [10.1–22.4]	99.0%	<0.0001	0.41
		Pyrazinamide	3	1296	18.9 [0.0–39.9]	98.0%	<0.0001	NA
	**Second line drugs**	Kanamycin	6	1251	0.5 [0.1–1.0]	5.0%	0.47	NA
		Amikacin	1	74	1.4 [0.0–4.0]	-	NA	NA
		Gatifloxacin	4	1165	0.2 [0.0–1.1]	44.0%	0.11	NA
		Ofloxacin	6	1674	7.3 [5.0–9.6]	53.0%	0.06	NA
		Moxifloxacin	2	999	5.8 [0.0–17.0]	89.0%	0.002	NA
		Ethionamide	1	74	35.1 [24.3–46.0]	-	NA	NA
		Prothionamide	2	904	15.7 [12.4–19.0]	48.0%	0.17	NA
		Cycloserine	1	74	44.6 [33.3–55.9]	-	NA	NA
		Para-aminosalicylic acid	1	74	12.2 [4.7–19.6]	-	NA	NA
		Levofloxacin	1	921	3.8 [2.6–5.0]	-	NA	NA
**Mono-DR**	**First line drugs**	Streptomycin	11	5090	6.1 [3.3–8.9]	94.0%	<0.0001	0.91
		Isoniazid	12	5199	4.2 [2.8–5.7]	88.0%	<0.0001	0.0004
		Rifampicin	12	5199	0.7 [0.3–1.1]	52.0%	0.02	0.0004
		Ethambutol	10	4990	0.8 [0.3–1.3]	64.0%	0.002	0.001
		Pyrazinamide	1	100	0.0 [0.0–1.4]	-	NA	NA

**Table 3:** Anti-tuberculosis-DR patterns in newly diagnosed and previously diagnosed tuberculosis patients.

Drug-Resistance Patterns		Antibiotics	Number of Analyzed Studies	Total Number of Tuberculosis Patients	Prevalence of Antibiotic-Resistance [95% CIs] (%)	Heterogeneity	
						*I* ^2^	*p*-Value
**Newly diagnosed tuberculosis patients**							
**Any-DR**	**First line drugs**	Streptomycin	4	2909	21.4 [12.9–29.9]	96.0%	<0.0001
		Isoniazid	4	2909	10.8 [6.2–15.4]	92.0%	<0.0001
		Rifampicin	4	2909	2.8 [0.6–5.1]	88.0%	<0.0001
		Ethambutol	4	2909	3.7 [1.0–6.5]	93.0%	<0.0001
		Pyrazinamide	1	751	2.8 [1.6–4.0]	-	NA
	**Second line drugs**	Gatifloxacin	2	779	0.0 [0.0–0.2]	0.0%	0.50
		Kanamycin	1	50	0.0 [0.0–2.7]	-	NA
		Levofloxacin	1	729	3.3 [2.0–4.6]	-	NA
		Moxifloxacin	1	732	0.4 [0.0–0.9]	-	NA
		Ofloxacin	2	786	5.7 [1.2–10.1]	39.0%	0.20
**Mono-DR**	**First line drugs**	Streptomycin	6	3505	9.3 [5.4–13.1]	91.0%	<0.0001
		Isoniazid	6	3505	2.9 [1.4–4.5]	83.0%	<0.0001
		Rifampicin	6	3505	0.3 [0.1–0.5]	8.0%	0.32
		Ethambutol	6	3505	0.3 [0.0–0.6]	58.0%	0.06
**Previously treated tuberculosis patients**							
**Any-DR**	**First line drugs**	Streptomycin	4	621	42.5 [13.8–71.3]	98.0%	<0.0001
		Isoniazid	5	727	49.9 [20.5–79.3]	99.0%	<0.0001
		Rifampicin	5	727	40.9 [10.8–71.0]	99.0%	<0.0001
		Ethambutol	4	621	27.6 [0.0–61.0]	99.0%	<0.0001
		Pyrazinamide	1	192	14.6 [9.6–19.6]	-	NA
	**Second line drugs**	Kanamycin	6	1251	0.5 [0.1–1.0]	5.0%	0.47
		Amikacin	1	74	1.4 [0.0–4.0]	-	NA
		Gatifloxacin	2	242	0.0 [0.0–0.7]	0.0%	0.61
		Ofloxacin	3	352	9.4 [6.3–12.4]	0.0%	0.98
		Moxifloxacin	2	266	6.2 [0.0–16.5]	86.0%	0.006
		Ethionamide	1	74	35.1 [24.3–46.0]	-	NA
		Prothionamide	2	904	15.7 [12.4–19.0]	48.0%	0.17
		Cycloserine	1	74	44.6 [33.3–55.9]	-	NA
		Para-aminosalicylic acid	1	74	12.2 [4.7–19.6]	-	NA
		Levofloxacin	1	192	5.2 [2.1–8.4]	-	NA
**Mono-DR**	**First line drugs**	Streptomycin	6	862	6.5 [3.9–9.1]	52.0%	0.06
		Isoniazid	7	971	3.5 [1.1–5.9]	84.0%	<0.0001
		Rifampicin	7	971	0.7 [0.1–1.2]	0.0%	0.77
		Ethambutol	6	862	0.7 [0.0–1.5]	36.0%	0.23
	**Second line drugs**	Ofloxacin	1	109	0.0 [0.0–1.3]	-	NA

**Table 4:** Sensitivity analyses.

Strategies of Sensitivity Analyses	Prevalence of Antibiotic Resistance [95% CIs] (%)	Difference of Pooled Prevalence Compared to the Main Result	Number of Studies Analyzed	Total Number of TB Patients	Heterogeneity	
					*I* ^2^	*p*-Value
**Any-DR**						
Excluding small studies	48.0 [34.2–61.8]	6.0% higher	12	6168	99.0%	<0.0001
Excluding low-quality studies	43.5 [31.7–55.3]	4.0% lower	15	6151	99.0%	<0.0001
**Mono-DR**						
Excluding small studies	14.1 [11.0–17.3]	1.4% lower	10	5234	90.0%	<0.0001
Excluding low-quality studies	14.3 [11.4–17.2]	0% change	13	5444	88.0%	<0.0001
**Multi-DR**						
Excluding small studies	22.9 [19.0–26.7]	3.2% higher	14	10,921	99.0%	<0.0001
Excluding low-quality studies	18.8 [14.7–23.0]	15.3% lower	16	6235	99.0%	<0.0001
**Poly-DR**						
Excluding small studies	7.7 [5.5–9.9]	0% change	8	5025	90.0%	<0.0001
Excluding low-quality studies	7.7 [5.6–9.7]	0% change	10	5235	88.0%	<0.0001
**Extensive-DR**						
Excluding small studies	0.1 [0.0–0.4]	66.7%	3	1051	0.0%	0.64
Excluding low-quality studies	0.1 [0.0–0.5]	66.7%	4	1135	11.0%	0.46

CIs: Confidence intervals.

## Supplementary Materials

The following are available online at https://www.mdpi.com/2079-6382/9/10/710/s1, Table S1: Search strategy, Table S2: Quality assessment of the included cross-sectional studies, Table S3: Quality assessment of the included cohort studies, Figure S1: Any resistance to first- and second-lines anti-TB drugs: (A) streptomycin, (B) isoniazid, (C) rifampicin, (D) ethambutol, (E) pyrazinamide, (F) kanamycin, (G) amikacin, (H) gatifloxacin, (I) ofloxacin, (J) moxifloxacin, (K) ethionamide, (L) prothionamide, (M) cycloserine, (N) para-aminosalicylic acid, and (O) levofloxacin, Figure S2: Mono resistance to anti-TB drugs: (A) streptomycin, (B) isoniazid, (C) rifampicin, (D) ethambutol, and (E) pyrazinamide, Figure S3: Any resistance to anti-TB drugs in newly diagnosed patients: (A) streptomycin (B) isoniazid, (C) rifampicin, (D) ethambutol (E) pyrazinamide, (F) gatifloxacin, (G) kanamycin, (H) levofloxacin, (I) moxifloxacin, (J) ofloxacin resistance and any resistance to anti-TB drugs in previously treated and newly diagnosed patients as well as any resistance to anti-TB drugs in previously treated patients: (K) streptomycin, (L) isoniazid, (M) rifampicin, (N) ethambutol, (O) pyrazinamide, (P) kanamycin, (Q) amikacin, (R) gatifloxacin, (S) ofloxacin, (T) moxifloxacin, (U) ethionamide, (V) prothionamide, (W) cycloserine, (X) para-aminosalicylic acid, and (Y) levofloxacin, Figure S4: Mono resistance to anti-TB drugs in newly diagnosed patients: (A) streptomycin, (B) isoniazid, (C) rifampicin, and (D) ethambutol and mono resistance to anti- TB drugs in previously treated patients: (E) streptomycin, (F) isoniazid, (G) rifampicin, (H) ethambutol, and (I) ofloxacin, Figure S5: Overall DRs in newly diagnosed and previously treated TB patients. (A) Any-DR in newly diagnosed patients, (B) any-DR in previously treated patients, (C) mono-DR in newly diagnosed patients, (D) mono-DR in previously treated patients, (E) multi-DR in newly diagnosed patients, (F) multi-DR in previously treated patients, (G) poly-DR in newly diagnosed patients, and (H) poly-DR in previously treated patients, Figure S6: Sensitivity analyses: excluding low-quality studies from (A) any drug-resistance, (B) multi drug-resistance, (C) extensive drug-resistance; excluding small studies from (D) any drug-resistance, (E) mono drug-resistance, (F) multi drug-resistance, (G) poly drug-resistance, and H) extensive drug-resistance.
