# Supplementary material for: Prevalence of Antibiotic-Resistant Pulmonary Tuberculosis in Bangladesh: A Systematic Review and Meta-Analysis
Source: Antibiotics (Basel). 2020 Oct 17;9(10):710. doi: 10.3390/antibiotics9100710 (PMC7602942; doi:10.3390/antibiotics9100710)
Supplement: Supplementary file 1 [file antibiotics-09-00710-s001.pdf]

Table S1: Search strategy

Databases	Search strategy
**PubMed**	(((((TB[Title] OR tuberculosis[Title] OR Mycobacterium tuberculosis[Title] OR anti-tuberculosis[Title] OR anti-tubercular)[Title])) AND ((isoniazid[Title] OR rifampin [Title] OR rifampicin [Title] OR rifamycin [Title] OR ethambutol [Title] OR pyrazinamide[Title] OR streptomycin[Title] OR amikacin[Title] OR kanamycin[Title] OR capreomycin[Title] OR viomycin[Title] OR enviomycin[Title] OR Ciprofloxacin[Title] OR Levofloxacin[Title] OR Moxifloxacin[Title] OR ethionamide[Title] OR prothionamide [Title] OR seromycin[Title] OR Terizidone[Title] OR Rifabutin[Title] OR clarithromycin[Title] OR Linezolid[Title] OR thioacetazone[Title] OR Bedaquiline[Title] OR Clofazimine[Title] OR rifapentine[Title] OR resistance*[Title] OR resistant*[Title] OR susceptibilit*[Title] OR sensitivit*)[Title]))) AND (((Bangladesh[Affiliation]) OR (Bangladesh[Title/Abstract] OR Dhaka[Title/Abstract] OR Chittagong[Title/Abstract] OR Chattogram[Title/Abstract] OR Rajshahi[Title/Abstract] OR Rangpur[Title/Abstract] OR Barisal[Title/Abstract] OR Barishal[Title/Abstract] OR Sylhet[Title/Abstract] OR Khulna[Title/Abstract] OR Mymensingh[Title/Abstract] OR Dinajpur[Title/Abstract] OR Bogra[Title/Abstract] OR Bogura[Title/Abstract] OR Comilla[Title/Abstract] OR Cumilla[Title/Abstract] OR Faridpur[Title/Abstract] OR Pabna[Title/Abstract] OR Noakhali[Title/Abstract] OR "Cox's Bazar"[Title/Abstract] OR Jessore[Title/Abstract] OR Jashore[Title/Abstract] OR Satkhira[Title/Abstract] OR Gazipur[Title/Abstract] OR Kushtia[Title/Abstract] OR Sirajganj[Title/Abstract] OR Gopalganj[Title/Abstract] OR Jamalpur[Title/Abstract] OR Gazipur[Title/Abstract] OR Tangail[Title/Abstract] OR Manikganj[Title/Abstract] OR Patuakhali[Title/Abstract] OR Rangamati[Title/Abstract] OR Chandpur[Title/Abstract] OR Netrakona[Title/Abstract] OR Magura[Title/Abstract] OR Naogaon[Title/Abstract] OR Nilphamari)[Title/Abstract]))
**Scopus**	TITLE(TB OR tuberculosis OR "Mycobacterium tuberculosis" OR anti-tuberculosis OR anti-tubercular) AND TITLE(isoniazid OR rifampin OR rifampicin OR rifamycin OR ethambutol OR pyrazinamide OR streptomycin OR amikacin OR kanamycin OR capreomycin OR viomycin OR enviomycin OR Ciprofloxacin OR Levofloxacin OR Moxifloxacin OR ethionamide OR prothionamide OR seromycin OR Terizidone OR Rifabutin OR clarithromycin OR Linezolid OR thioacetazone OR Bedaquiline OR Clofazimine OR rifapentine OR resistance* OR resistant* OR susceptibilit* OR sensitivit*) AND AFFIL(Bangladesh) OR TITLE(Bangladesh OR Dhaka OR Chittagong OR Chattogram OR Rajshahi OR Rangpur OR Barisal OR Barishal OR Sylhet OR Khulna OR Mymensingh OR Dinajpur OR Bogra OR Bogura OR Comilla OR Cumilla OR Faridpur OR Pabna OR Noakhali OR "Cox's Bazar" OR Jessore OR Jashore OR Satkhira OR Gazipur OR Kushtia OR Sirajganj OR Gopalganj OR Jamalpur OR Gazipur OR Tangail OR Manikganj OR Patuakhali OR Rangamati OR Chandpur OR Netrakona OR Magura OR Naogaon OR Nilphamari)
**Google Scholar**	allintitle: (TB OR tuberculosis OR "Mycobacterium tuberculosis" OR anti-tuberculosis OR anti-tubercular) (Bangladesh OR Dhaka OR Chittagong OR Chattogram OR Rajshahi OR Rangpur OR Barisal OR Barishal OR Sylhet OR Khulna OR Mymensingh OR Dinajpur OR Bogra OR Comilla OR Cumilla OR Faridpur OR Pabna OR Noakhali OR "Cox's Bazar" OR Jessore OR Jashore OR Satkhira OR Gazipur OR Kushtia OR Sirajganj OR Gopalganj OR Jamalpur OR Gazipur OR Tangail OR Manikganj OR Patuakhali OR Rangamati OR Chandpur OR Netrakona OR Magura OR Naogaon OR Nilphamari)

Table S2: Quality assessment of the included cross-sectional studies

No	Study ID	JBI critical appraisal tool								Yes (%)
		1	2	3	4	5	6	7	8	
1	Aurin 2014	Y	Y	Y	Y	N	N	N	N	50.0
2	Banu 2010	Y	Y	Y	Y	Y	Y	Y	Y	100.0
3	Banu 2012	Y	Y	Y	Y	Y	Y	Y	Y	100.0
4	Banu 2013	Y	Y	Y	Y	Y	Y	Y	Y	100.0
5	Banu 2017	Y	Y	Y	Y	Y	Y	Y	Y	100.0
6	Hussain 2005	Y	Y	Y	N	N	N	Y	N	50.0
7	Iqbal 2013	Y	Y	U	Y	N	N	U	N	37.5
8	Kamal 2015	Y	Y	Y	Y	Y	Y	Y	Y	100.0
9	Khatun 2017	Y	Y	Y	Y	N	N	Y	N	62.5
10	Mohiuddin 2014	Y	Y	Y	Y	N	N	Y	N	62.5
11	Mottalib 2011	Y	Y	Y	Y	N	N	Y	N	62.5
12	Noor 2012	Y	Y	Y	Y	N	N	Y	N	62.5
13	Noor 2013	Y	Y	Y	Y	N	N	Y	N	62.5
14	Rahman 2009	Y	Y	Y	Y	N	N	U	U	50.0
15	Storla 2007	Y	Y	Y	Y	N	N	Y	N	62.5
16	Uddin 2018	Y	Y	Y	Y	N	N	Y	Y	75.0
17	Van Deun 1999	Y	Y	Y	Y	N	N	Y	N	62.5
18	Wadud 2009	Y	Y	Y	Y	N	N	Y	N	62.5
19	Zaman 2005	Y	Y	Y	Y	Y	Y	Y	Y	100.0
20	Zignol 2016	Y	Y	Y	Y	U	Y	Y	Y	87.5

JBI: Joanna Briggs Institute, Y: Yes, N: No, U: Unclear.

Table S3: Quality assessment of the included cohort studies

No	Study ID	JBI critical appraisal tool											Yes (%)
		1	2	3	4	5	6	7	8	9	10	11	
1	Afroz 2013	Y	Y	Y	Y	Y	Y	Y	Y	Y	NA	Y	90.9
2	Aung 2014	Y	Y	Y	Y	Y	Y	Y	Y	N	N	Y	81.8
3	Heysell 2015	Y	Y	Y	Y	Y	Y	Y	Y	N	Y	Y	90.9
4	Van Deun 2010	Y	Y	Y	N	N	Y	N	Y	N	N	Y	54.5

JBI: Joanna Briggs Institute, Y: Yes, N: No, NA: Not applicable.

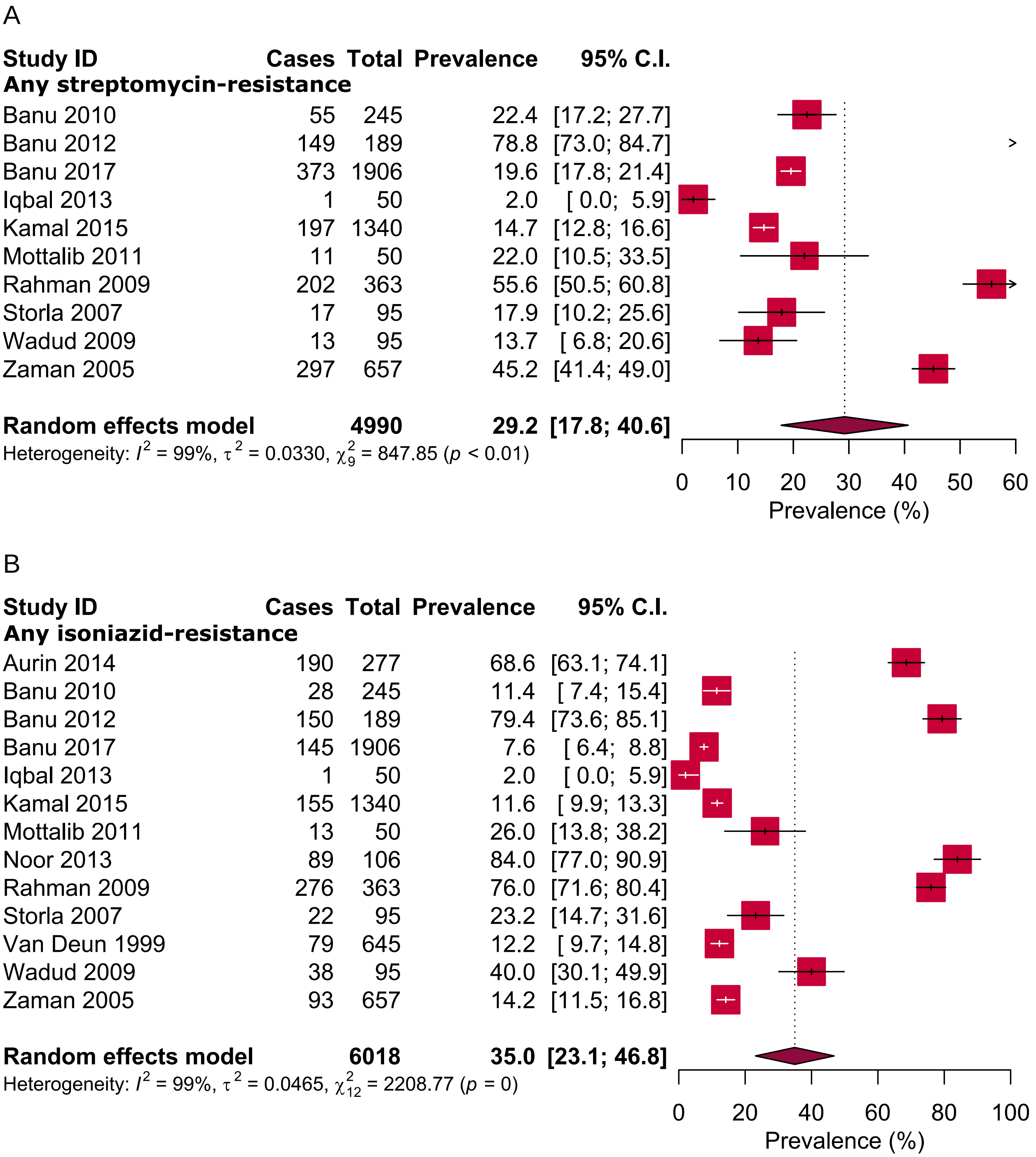
A
Study ID Cases Total Prevalence 95% C.I.
Any streptomycin-resistance
Banu 2010 55 245 22.4 [17.2; 27.7]
Banu 2012 149 189 78.8 [73.0; 84.7]
Banu 2017 373 1906 19.6 [17.8; 21.4]
Iqbal 2013 1 50 2.0 [0.0; 5.9]
Kamal 2015 197 1340 14.7 [12.8; 16.6]
Mottalib 2011 11 50 22.0 [10.5; 33.5]
Rahman 2009 202 363 55.6 [50.5; 60.8]
Storla 2007 17 95 17.9 [10.2; 25.6]
Wadud 2009 13 95 13.7 [6.8; 20.6]
Zaman 2005 297 657 45.2 [41.4; 49.0]
Random effects model 4990 29.2 [17.8; 40.6]
Heterogeneity: I^2 = 99%, τ^2 = 0.0330, χ^2_9 = 847.85 (p < 0.01)
0 10 20 30 40 50 60
Prevalence (%)
B
Study ID Cases Total Prevalence 95% C.I.
Any isoniazid-resistance
Aurin 2014 190 277 68.6 [63.1; 74.1]
Banu 2010 28 245 11.4 [7.4; 15.4]
Banu 2012 150 189 79.4 [73.6; 85.1]
Banu 2017 145 1906 7.6 [6.4; 8.8]
Iqbal 2013 1 50 2.0 [0.0; 5.9]
Kamal 2015 155 1340 11.6 [9.9; 13.3]
Mottalib 2011 13 50 26.0 [13.8; 38.2]
Noor 2013 89 106 84.0 [77.0; 90.9]
Rahman 2009 276 363 76.0 [71.6; 80.4]
Storla 2007 22 95 23.2 [14.7; 31.6]
Van Deun 1999 79 645 12.2 [9.7; 14.8]
Wadud 2009 38 95 40.0 [30.1; 49.9]
Zaman 2005 93 657 14.2 [11.5; 16.8]
Random effects model 6018 35.0 [23.1; 46.8]
Heterogeneity: I^2 = 99%, τ^2 = 0.0465, χ^2_{12} = 2208.77 (p = 0)
0 20 40 60 80 100
Prevalence (%)

C

Study ID	Cases	Total	Prevalence	95% C.I.
**Any rifampicin-resistance**				
Aurin 2014	188	277	67.9	[62.4; 73.4]
Banu 2010	2	245	0.8	[0.0; 1.9]
Banu 2012	146	189	77.2	[71.3; 83.2]
Banu 2017	74	1906	3.9	[3.0; 4.7]
Iqbal 2013	1	50	2.0	[0.0; 5.9]
Kamal 2015	98	1340	7.3	[5.9; 8.7]
Mottalib 2011	6	50	12.0	[3.0; 21.0]
Noor 2013	84	106	79.2	[71.5; 87.0]
Rahman 2009	260	363	71.6	[67.0; 76.3]
Storla 2007	2	95	2.1	[0.0; 5.0]
Van Deun 1999	18	645	2.8	[1.5; 4.1]
Wadud 2009	31	95	32.6	[23.2; 42.1]
Zaman 2005	42	657	6.4	[4.5; 8.3]
**Random effects model**		**6018**	**27.6**	**[19.9; 35.4]**

Heterogeneity: $I^2 = 99\%$, $\tau^2 = 0.0197$, $\chi^2_{12} = 2322.82$ ($p = 0$)

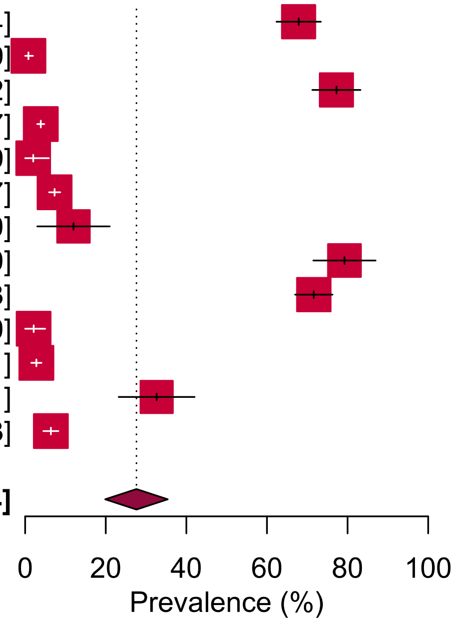

D

Study ID	Cases	Total	Prevalence	95% C.I.
**Any ethambutol-resistance**				
Banu 2010	16	245	6.5	[3.4; 9.6]
Banu 2012	145	189	76.7	[70.7; 82.7]
Banu 2017	68	1906	3.6	[2.7; 4.4]
Iqbal 2013	0	50	0.0	[0.0; 2.7]
Kamal 2015	59	1340	4.4	[3.3; 5.5]
Mottalib 2011	10	50	20.0	[8.9; 31.1]
Rahman 2009	100	363	27.5	[23.0; 32.1]
Storla 2007	9	95	9.5	[3.6; 15.4]
Wadud 2009	11	95	11.6	[5.1; 18.0]
Zaman 2005	52	657	7.9	[5.9; 10.0]
**Random effects model**		**4990**	**16.2**	**[10.1; 22.4]**

Heterogeneity: $I^2 = 99\%$, $\tau^2 = 0.0093$, $\chi^2_{9} = 682.79$ ($p < 0.01$)

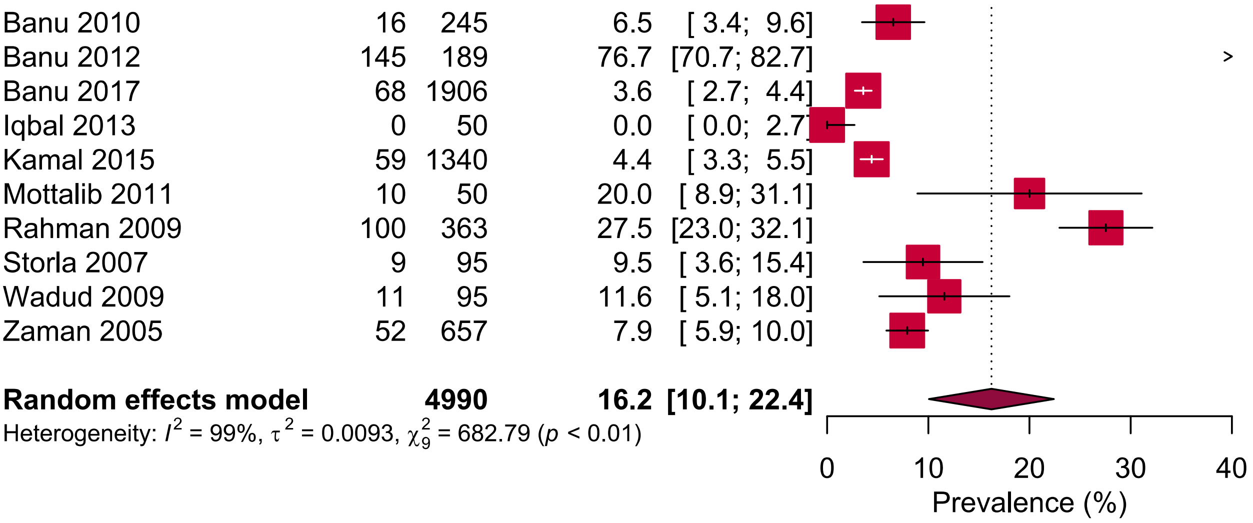

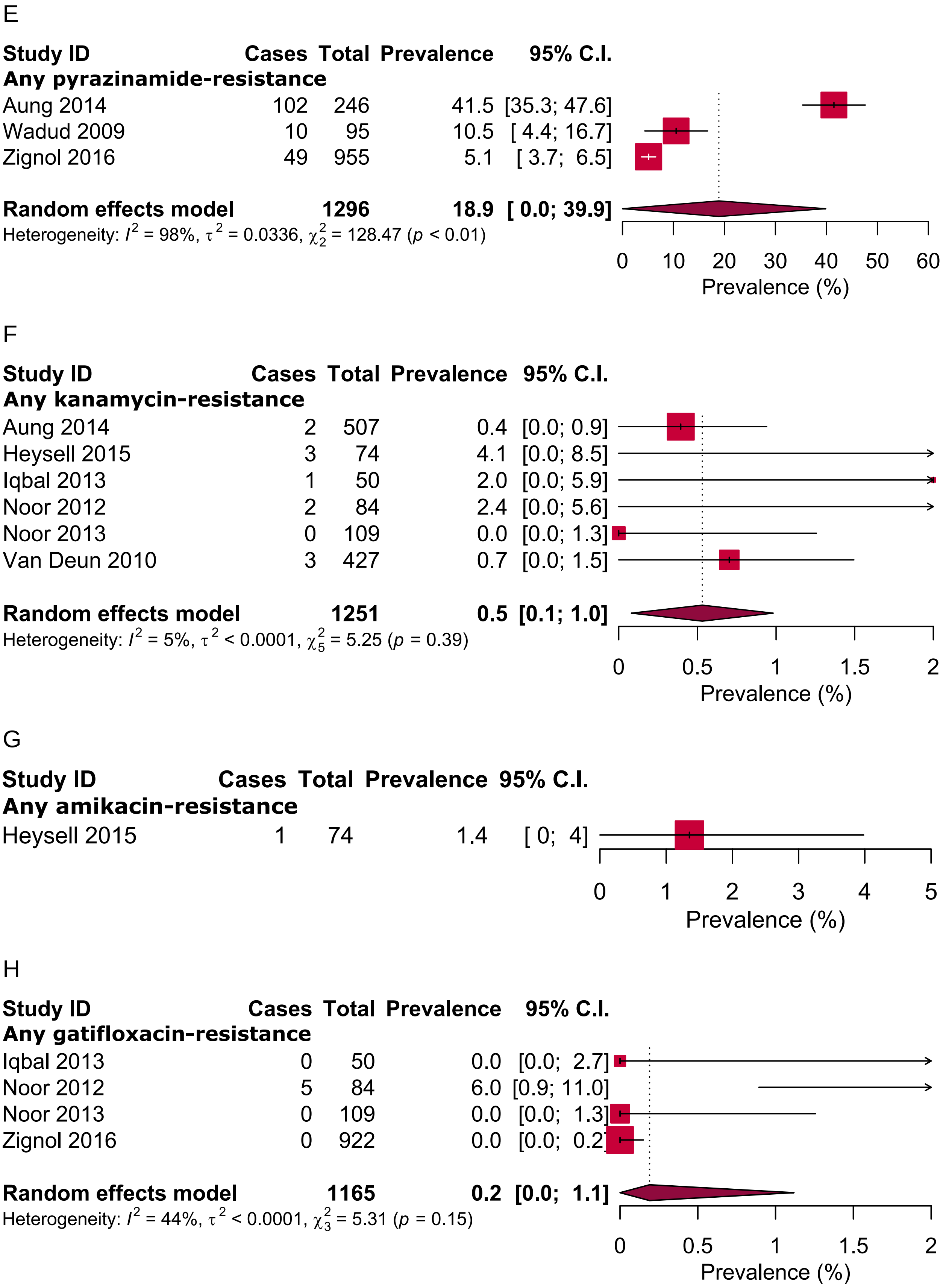
E
Study ID Cases Total Prevalence 95% C.I.
Any pyrazinamide-resistance
Aung 2014 102 246 41.5 [35.3; 47.6]
Wadud 2009 10 95 10.5 [4.4; 16.7]
Zignol 2016 49 955 5.1 [3.7; 6.5]
Random effects model 1296 18.9 [0.0; 39.9]
Heterogeneity: I^2 = 98%, τ^2 = 0.0336, χ^2_2 = 128.47 (p < 0.01)
0 10 20 30 40 50 60
Prevalence (%)
F
Study ID Cases Total Prevalence 95% C.I.
Any kanamycin-resistance
Aung 2014 2 507 0.4 [0.0; 0.9]
Heysell 2015 3 74 4.1 [0.0; 8.5]
Iqbal 2013 1 50 2.0 [0.0; 5.9]
Noor 2012 2 84 2.4 [0.0; 5.6]
Noor 2013 0 109 0.0 [0.0; 1.3]
Van Deun 2010 3 427 0.7 [0.0; 1.5]
Random effects model 1251 0.5 [0.1; 1.0]
Heterogeneity: I^2 = 5%, τ^2 < 0.0001, χ^2_5 = 5.25 (p = 0.39)
0 0.5 1 1.5 2
Prevalence (%)
G
Study ID Cases Total Prevalence 95% C.I.
Any amikacin-resistance
Heysell 2015 1 74 1.4 [0; 4]
0 1 2 3 4 5
Prevalence (%)
H
Study ID Cases Total Prevalence 95% C.I.
Any gatifloxacin-resistance
Iqbal 2013 0 50 0.0 [0.0; 2.7]
Noor 2012 5 84 6.0 [0.9; 11.0]
Noor 2013 0 109 0.0 [0.0; 1.3]
Zignol 2016 0 922 0.0 [0.0; 0.2]
Random effects model 1165 0.2 [0.0; 1.1]
Heterogeneity: I^2 = 44%, τ^2 < 0.0001, χ^2_3 = 5.31 (p = 0.15)
0 0.5 1 1.5 2
Prevalence (%)

I

Study ID	Cases	Total	Prevalence	95% C.I.
**Any ofloxacin-resistance**				
Heysell 2015	13	74	17.6	[8.9; 26.2]
Iqbal 2013	5	50	10.0	[1.7; 18.3]
Noor 2012	7	84	8.3	[2.4; 14.2]
Noor 2013	10	106	9.4	[3.9; 15.0]
Van Deun 2010	23	427	5.4	[3.2; 7.5]
Zignol 2016	51	933	5.5	[4.0; 6.9]
**Random effects model**		**1674**	**7.3**	**[5.0; 9.6]**

Heterogeneity: I^2 = 53%, τ^2 = 0.0003, χ^2_5 = 10.62 (p = 0.06)

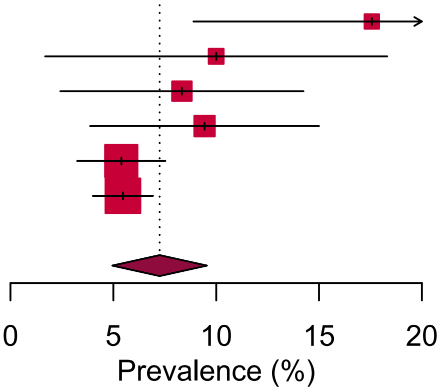

J

Study ID	Cases	Total	Prevalence	95% C.I.
**Any moxifloxacin-resistance**				
Heysell 2015	9	74	12.2	[4.7; 19.6]
Zignol 2016	6	925	0.6	[0.1; 1.2]
**Random effects model**		**999**	**5.8**	**[0.0; 17.0]**

Heterogeneity: I^2 = 89%, τ^2 = 0.0059, χ^2_1 = 9.14 (p < 0.01)

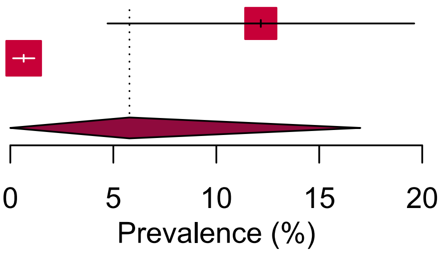

K

Study ID	Cases	Total	Prevalence	95% C.I.
**Any ethionamide-resistance**				
Heysell 2015	26	74	35.1	[24.3; 46]

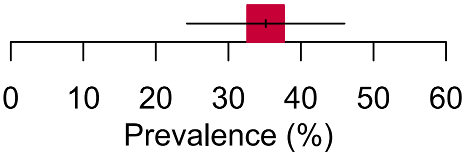

L

Study ID	Cases	Total	Prevalence	95% C.I.
**Any prothionamide-resistance**				
Aung 2014	83	477	17.4	[14.0; 20.8]
Van Deun 2010	60	427	14.1	[10.8; 17.3]
**Random effects model**		**904**	**15.7**	**[12.4; 19.0]**

Heterogeneity: I^2 = 48%, τ^2 = 0.0003, χ^2_1 = 1.92 (p = 0.17)

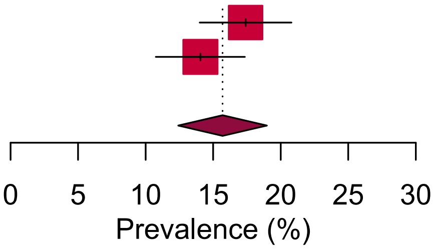

M

Study ID	Cases	Total	Prevalence	95% C.I.
**Any cycloserine-resistance**				
Heysell 2015	33	74	44.6	[33.3; 55.9]

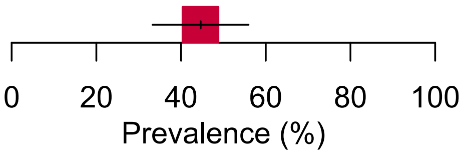

N

Study ID	Cases	Total	Prevalence	95% C.I.
**Any para-aminosalicylic acid-resistance**				
Heysell 2015	9	74	12.2	[4.7; 19.6]

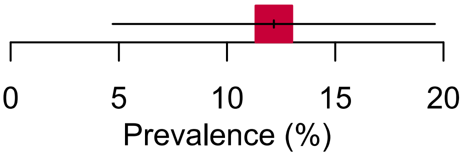

O

Study ID	Cases	Total	Prevalence	95% C.I.
**Any levofloxacin-resistance**				
Zignol 2016	35	921	3.8	[2.6; 5]

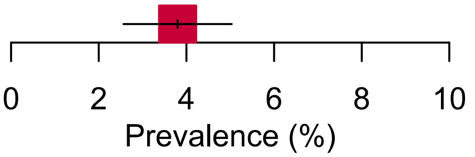

Figure S1: Any resistance to first- and second-lines anti-TB drugs: A) streptomycin, B) isoniazid, C) rifampicin, D) ethambutol, E) pyrazinamide, F) kanamycin, G) amikacin, H) gatifloxacin, I) ofloxacin, J) moxifloxacin, K) ethionamide, L) prothionamide, M) cycloserine, N) para-aminosalicylic acid, and O) levofloxacin.

A

Study ID	Cases	Total	Prevalence	95% C.I.
**Mono streptomycin-resistance**				
Afroz 2013	4	264	1.5	[0.0; 3.0]
Banu 2012	10	189	5.3	[2.1; 8.5]
Banu 2013	1	20	5.0	[0.0; 14.6]
Banu 2017	255	1906	13.4	[11.9; 14.9]
Kamal 2015	90	1340	6.7	[5.4; 8.1]
Khatun 2017	4	100	4.0	[0.2; 7.8]
Mohiuddin 2014	24	192	12.5	[7.8; 17.2]
Storla 2007	6	95	6.3	[1.4; 11.2]
Uddin 2018	16	244	6.6	[3.5; 9.7]
Van Deun 1999	33	645	5.1	[3.4; 6.8]
Wadud 2009	1	95	1.1	[0.0; 3.1]
**Random effects model**		**5090**	**6.1**	**[3.3; 8.9]**

Heterogeneity: $I^2 = 94\%$, $\tau^2 = 0.0019$, $\chi^2_{10} = 158.38$ ($p < 0.01$)

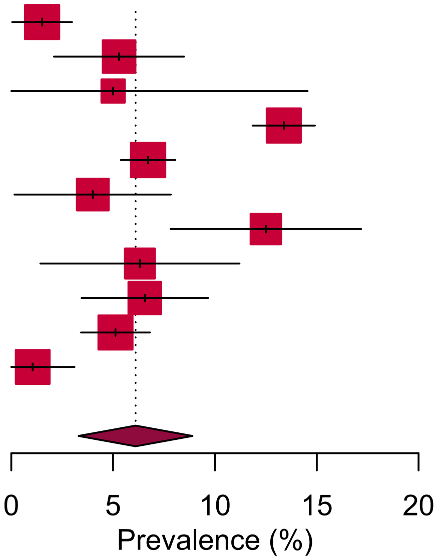

B

Study ID	Cases	Total	Prevalence	95% C.I.
**Mono isoniazid-resistance**				
Afroz 2013	13	264	4.9	[2.3; 7.5]
Banu 2012	1	189	0.5	[0.0; 1.6]
Banu 2013	0	20	0.0	[0.0; 6.5]
Banu 2017	20	1906	1.0	[0.6; 1.5]
Kamal 2015	21	1340	1.6	[0.9; 2.2]
Khatun 2017	13	100	13.0	[6.4; 19.6]
Mohiuddin 2014	18	192	9.4	[5.3; 13.5]
Noor 2013	9	109	8.3	[3.1; 13.4]
Storla 2007	9	95	9.5	[3.6; 15.4]
Uddin 2018	6	244	2.5	[0.5; 4.4]
Van Deun 1999	44	645	6.8	[4.9; 8.8]
Wadud 2009	10	95	10.5	[4.4; 16.7]
**Random effects model**		**5199**	**4.2**	**[2.8; 5.7]**

Heterogeneity: $I^2 = 88\%$, $\tau^2 = 0.0004$, $\chi^2_{11} = 90.99$ ($p < 0.01$)

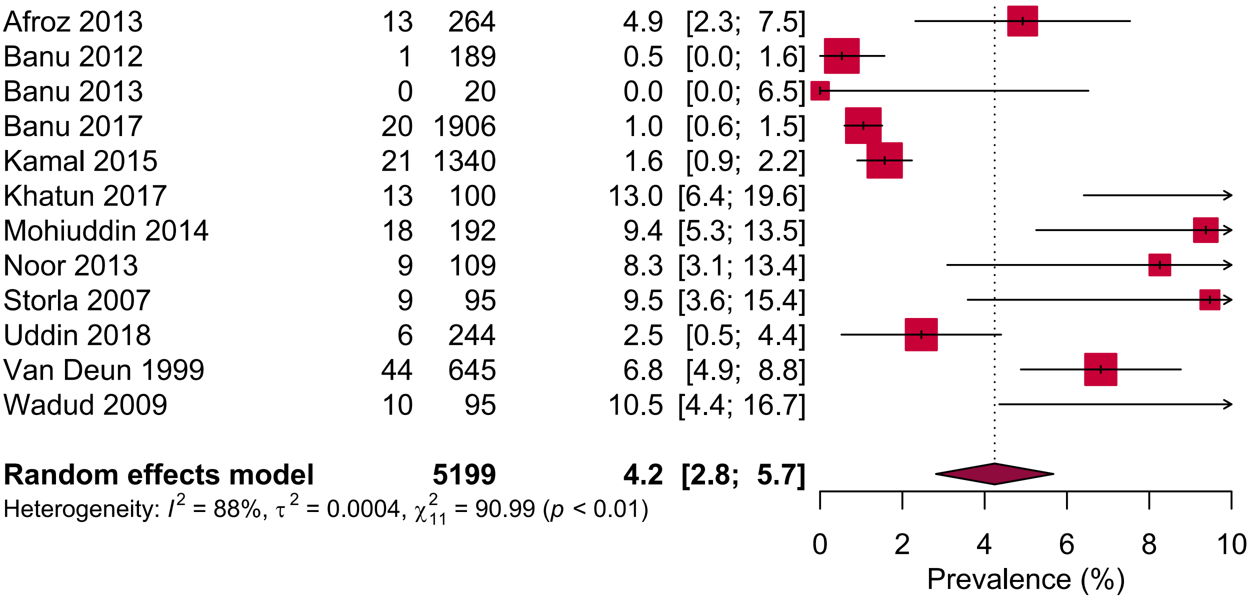

C

Study ID	Cases	Total	Prevalence	95% C.I.
**Mono rifampicin-resistance**				
Afroz 2013	9	264	3.4	[1.2; 5.6]
Banu 2012	2	189	1.1	[0.0; 2.5]
Banu 2013	0	20	0.0	[0.0; 6.5]
Banu 2017	9	1906	0.5	[0.2; 0.8]
Kamal 2015	4	1340	0.3	[0.0; 0.6]
Khatun 2017	2	100	2.0	[0.0; 4.7]
Mohiuddin 2014	5	192	2.6	[0.4; 4.9]
Noor 2013	2	109	1.8	[0.0; 4.4]
Storla 2007	0	95	0.0	[0.0; 1.4]
Uddin 2018	1	244	0.4	[0.0; 1.2]
Van Deun 1999	3	645	0.5	[0.0; 1.0]
Wadud 2009	8	95	8.4	[2.8; 14.0]
**Random effects model**		**5199**	**0.7**	**[0.3; 1.1]**

Heterogeneity: $I^2 = 52\%$, $\tau^2 < 0.0001$, $\chi^2_{11} = 22.87$ ($p = 0.02$)

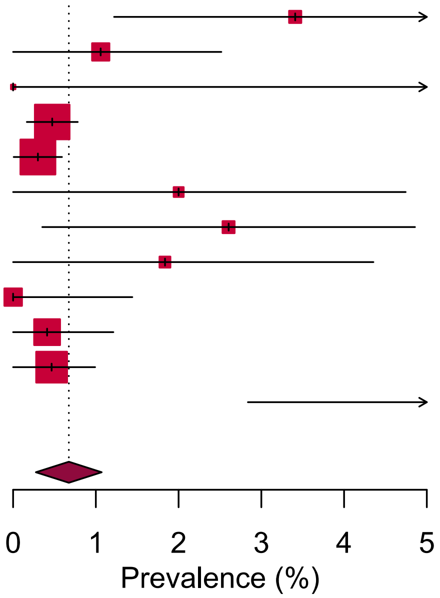

D

Study ID	Cases	Total	Prevalence	95% C.I.
**Mono ethambutol-resistance**				
Afroz 2013	5	264	1.9	[0.2; 3.5]
Banu 2012	4	189	2.1	[0.1; 4.2]
Banu 2013	0	20	0.0	[0.0; 6.5]
Banu 2017	7	1906	0.4	[0.1; 0.6]
Kamal 2015	3	1340	0.2	[0.0; 0.5]
Mohiuddin 2014	10	192	5.2	[2.1; 8.4]
Storla 2007	1	95	1.1	[0.0; 3.1]
Uddin 2018	8	244	3.3	[1.0; 5.5]
Van Deun 1999	3	645	0.5	[0.0; 1.0]
Wadud 2009	2	95	2.1	[0.0; 5.0]
**Random effects model**		**4990**	**0.8**	**[0.3; 1.3]**

Heterogeneity: $I^2 = 64\%$, $\tau^2 < 0.0001$, $\chi^2_{9} = 24.91$ ($p < 0.01$)

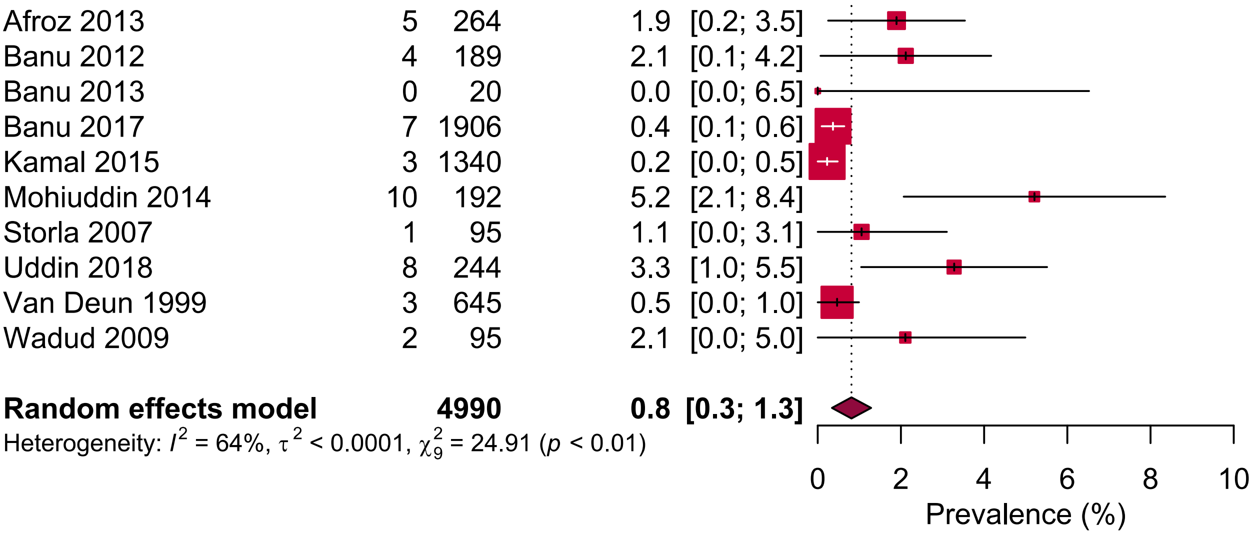

E

Study ID	Cases	Total	Prevalence	95% C.I.
**Mono pyrazinamide-resistance**				
Khatun 2017	0	100	0.0	[0; 1.4]

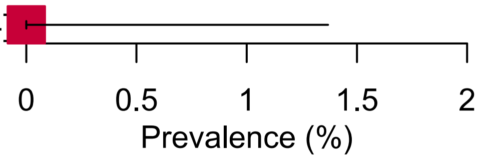

Figure S2: Mono resistance to anti-TB drugs: A) streptomycin, B) isoniazid, C) rifampicin, D) ethambutol, and E) pyrazinamide.

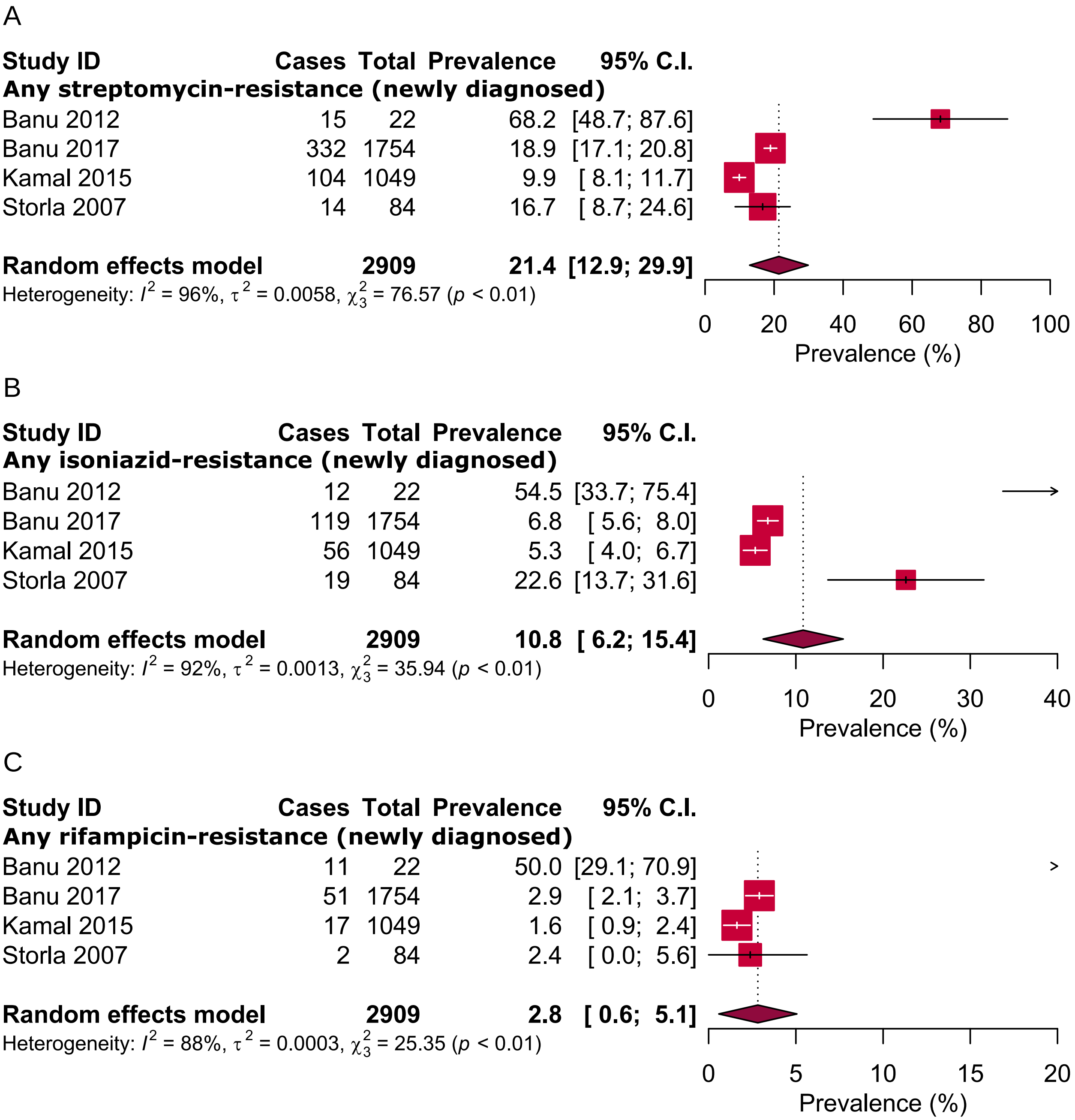
A
Study ID Cases Total Prevalence 95% C.I.
Any streptomycin-resistance (newly diagnosed)
Banu 2012 15 22 68.2 [48.7; 87.6]
Banu 2017 332 1754 18.9 [17.1; 20.8]
Kamal 2015 104 1049 9.9 [8.1; 11.7]
Storla 2007 14 84 16.7 [8.7; 24.6]
Random effects model 2909 21.4 [12.9; 29.9]
Heterogeneity: I^2 = 96%, τ^2 = 0.0058, χ^2_3 = 76.57 (p < 0.01)
0 20 40 60 80 100
Prevalence (%)
B
Study ID Cases Total Prevalence 95% C.I.
Any isoniazid-resistance (newly diagnosed)
Banu 2012 12 22 54.5 [33.7; 75.4]
Banu 2017 119 1754 6.8 [5.6; 8.0]
Kamal 2015 56 1049 5.3 [4.0; 6.7]
Storla 2007 19 84 22.6 [13.7; 31.6]
Random effects model 2909 10.8 [6.2; 15.4]
Heterogeneity: I^2 = 92%, τ^2 = 0.0013, χ^2_3 = 35.94 (p < 0.01)
0 10 20 30 40
Prevalence (%)
C
Study ID Cases Total Prevalence 95% C.I.
Any rifampicin-resistance (newly diagnosed)
Banu 2012 11 22 50.0 [29.1; 70.9]
Banu 2017 51 1754 2.9 [2.1; 3.7]
Kamal 2015 17 1049 1.6 [0.9; 2.4]
Storla 2007 2 84 2.4 [0.0; 5.6]
Random effects model 2909 2.8 [0.6; 5.1]
Heterogeneity: I^2 = 88%, τ^2 = 0.0003, χ^2_3 = 25.35 (p < 0.01)
0 5 10 15 20
Prevalence (%)

D

Study ID	Cases	Total	Prevalence	95% C.I.
**Any ethambutol-resistance (newly diagnosed)**				
Banu 2012	10	22	45.5	[24.6; 66.3]
Banu 2017	51	1754	2.9	[2.1; 3.7]
Kamal 2015	9	1049	0.9	[0.3; 1.4]
Storla 2007	9	84	10.7	[4.1; 17.3]
**Random effects model**		**2909**	**3.7**	**[1.0; 6.5]**

Heterogeneity: I^2 = 93%, τ^2 = 0.0005, χ^2_3 = 41.77 (p < 0.01)

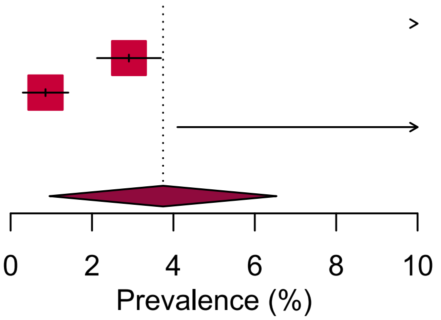

E

Study ID	Cases	Total	Prevalence	95% C.I.
**Any pyrazinamide-resistance (newly diagnosed)**				
Zignol 2016	21	751	2.8	[1.6; 4]

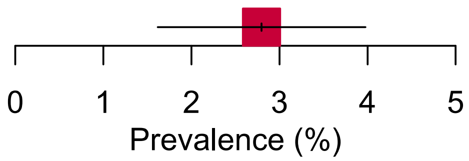

F

Study ID	Cases	Total	Prevalence	95% C.I.
**Any gatifloxacin-resistance (newly diagnosed)**				
Iqbal 2013	0	50	0.0	[0; 2.7]
Zignol 2016	0	729	0.0	[0; 0.2]
**Random effects model**		**779**	**0.0**	**[0; 0.2]**

Heterogeneity: I^2 = 0%, τ^2 = 0, χ^2_1 = 0.00 (p = 1.00)

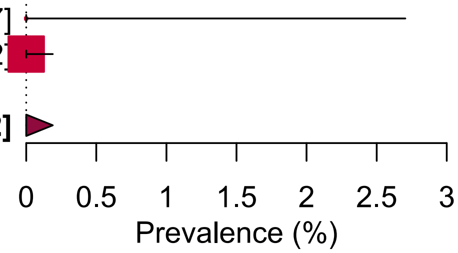

G

Study ID	Cases	Total	Prevalence	95% C.I.
**Any kanamycin-resistance (newly diagnosed)**				
Iqbal 2013	0	50	0.0	[0; 2.7]

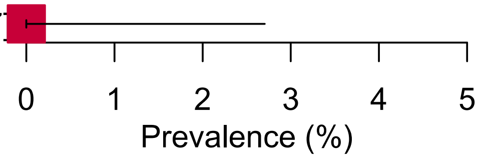

H

Study ID	Cases	Total	Prevalence	95% C.I.
**Any levofloxacin-resistance (newly diagnosed)**				
Zignol 2016	24	729	3.3	[2; 4.6]

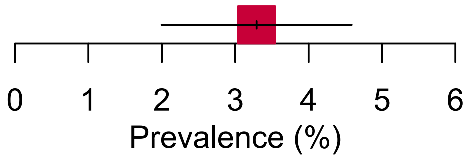

I

Study ID	Cases	Total	Prevalence	95% C.I.
**Any moxifloxacin-resistance (newly diagnosed)**				
Zignol 2016	3	732	0.4	[0; 0.9]

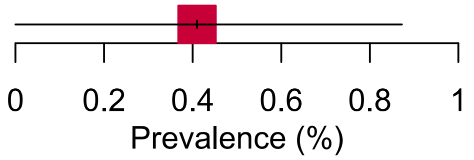

J

Study ID	Cases	Total	Prevalence	95% C.I.
**Any ofloxacin-resistance (newly diagnosed)**				
Iqbal 2013	5	50	10.0	[1.7; 18.3]
Zignol 2016	33	736	4.5	[3.0; 6.0]
**Random effects model**		**786**	**5.7**	**[1.2; 10.1]**

Heterogeneity: $I^2 = 39\%$, $\tau^2 = 0.0006$, $\chi^2_1 = 1.64$ ($p = 0.20$)

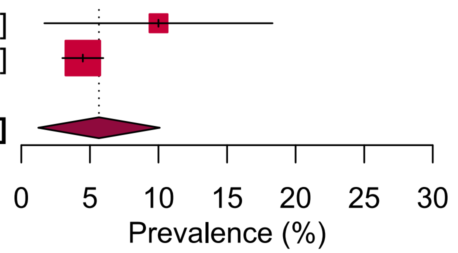

K

Study ID	Cases	Total	Prevalence	95% C.I.
**Any streptomycin-resistance (previously treated)**				
Banu 2012	134	167	80.2	[74.2; 86.3]
Banu 2017	41	152	27.0	[19.9; 34.0]
Kamal 2015	96	291	33.0	[27.6; 38.4]
Storla 2007	3	11	27.3	[1.0; 53.6]
**Random effects model**		**621**	**42.5**	**[13.8; 71.3]**

Heterogeneity: $I^2 = 98\%$, $\tau^2 = 0.0813$, $\chi^2_3 = 175.31$ ($p < 0.01$)

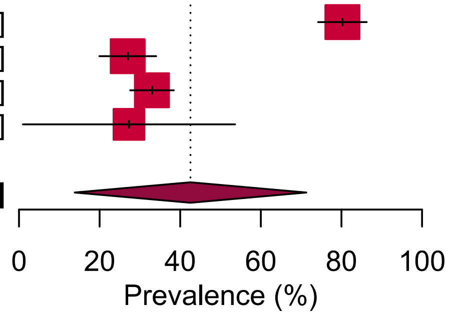

L

Study ID	Cases	Total	Prevalence	95% C.I.
**Any isoniazid-resistance (previously treated)**				
Banu 2012	138	167	82.6	[76.9; 88.4]
Banu 2017	26	152	17.1	[11.1; 23.1]
Kamal 2015	104	291	35.7	[30.2; 41.2]
Storla 2007	3	11	27.3	[1.0; 53.6]
Noor 2013	89	106	84.0	[77.0; 90.9]
**Random effects model**		**727**	**49.9**	**[20.5; 79.3]**

Heterogeneity: I^2 = 99%, τ^2 = 0.1084, χ^2_4 = 357.02 (p < 0.01)

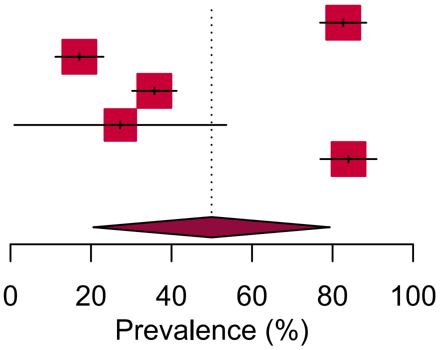

M

Study ID	Cases	Total	Prevalence	95% C.I.
**Any rifampicin-resistance (previously treated)**				
Banu 2012	135	167	80.8	[74.9; 86.8]
Banu 2017	23	152	15.1	[9.4; 20.8]
Kamal 2015	84	291	28.9	[23.7; 34.1]
Storla 2007	0	11	0.0	[0.0; 11.3]
Noor 2013	84	106	79.2	[71.5; 87.0]
**Random effects model**		**727**	**40.9**	**[10.8; 71.0]**

Heterogeneity: I^2 = 99%, τ^2 = 0.1165, χ^2_4 = 414.76 (p < 0.01)

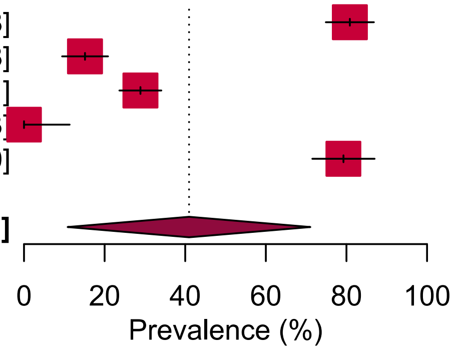

N

Study ID	Cases	Total	Prevalence	95% C.I.
**Any ethambutol-resistance (previously treated)**				
Banu 2012	135	167	80.8	[74.9; 86.8]
Banu 2017	17	152	11.2	[6.2; 16.2]
Kamal 2015	52	291	17.9	[13.5; 22.3]
Storla 2007	0	11	0.0	[0.0; 11.3]
**Random effects model**		**621**	**27.6**	**[0.0; 61.0]**

Heterogeneity: I^2 = 99%, τ^2 = 0.1148, χ^2_3 = 388.03 (p < 0.01)

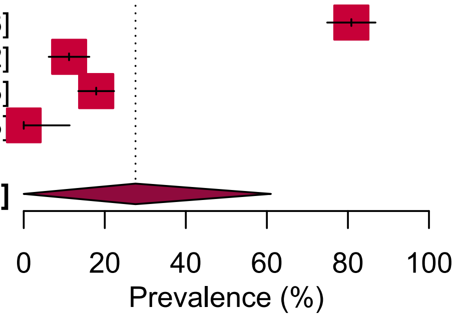

O

Study ID	Cases	Total	Prevalence	95% C.I.
**Any pyrazinamide-resistance (previously treated)**				
Zignol 2016	28	192	14.6	[9.6; 19.6]

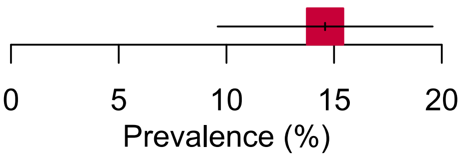

P

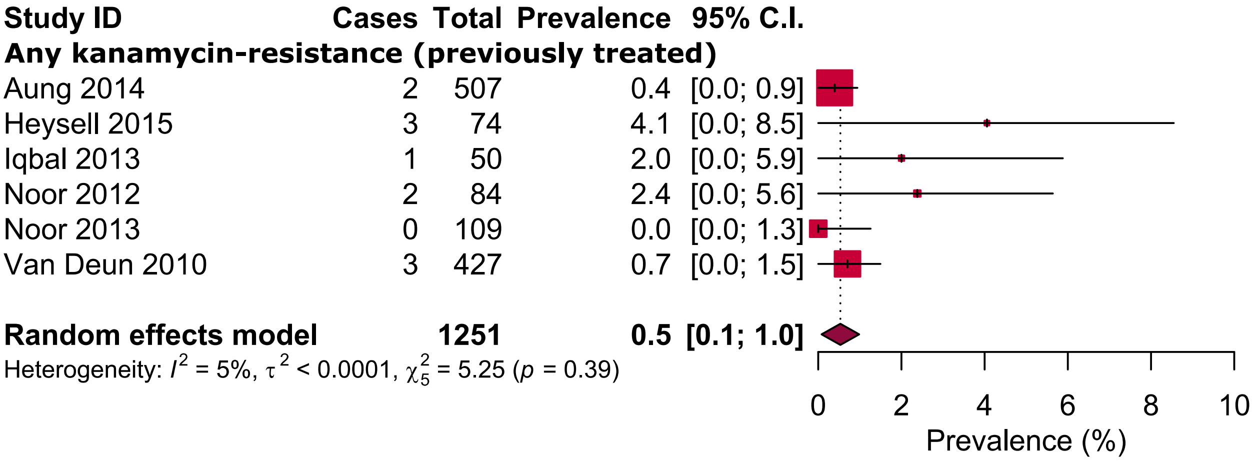
Study ID Cases Total Prevalence 95% C.I.
Any kanamycin-resistance (previously treated)
Aung 2014 2 507 0.4 [0.0; 0.9]
Heysell 2015 3 74 4.1 [0.0; 8.5]
Iqbal 2013 1 50 2.0 [0.0; 5.9]
Noor 2012 2 84 2.4 [0.0; 5.6]
Noor 2013 0 109 0.0 [0.0; 1.3]
Van Deun 2010 3 427 0.7 [0.0; 1.5]
Random effects model 1251 0.5 [0.1; 1.0]
Heterogeneity: I^2 = 5%, τ^2 < 0.0001, χ^2_5 = 5.25 (p = 0.39)
0 2 4 6 8 10
Prevalence (%)

Q

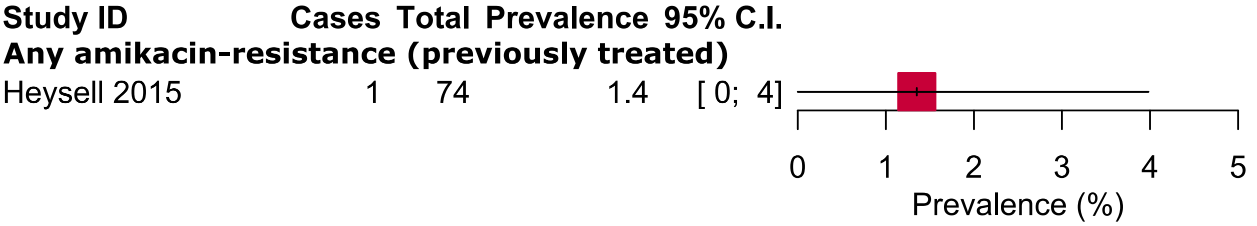
Study ID Cases Total Prevalence 95% C.I.
Any amikacin-resistance (previously treated)
Heysell 2015 1 74 1.4 [0; 4]
0 1 2 3 4 5
Prevalence (%)

R

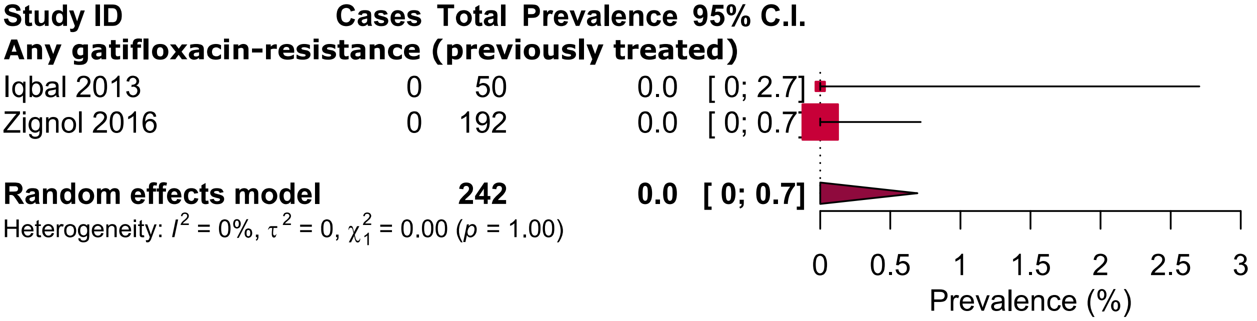
Study ID Cases Total Prevalence 95% C.I.
Any gatifloxacin-resistance (previously treated)
Iqbal 2013 0 50 0.0 [0; 2.7]
Zignol 2016 0 192 0.0 [0; 0.7]
Random effects model 242 0.0 [0; 0.7]
Heterogeneity: I^2 = 0%, τ^2 = 0, χ^2_1 = 0.00 (p = 1.00)
0 0.5 1 1.5 2 2.5 3
Prevalence (%)

S

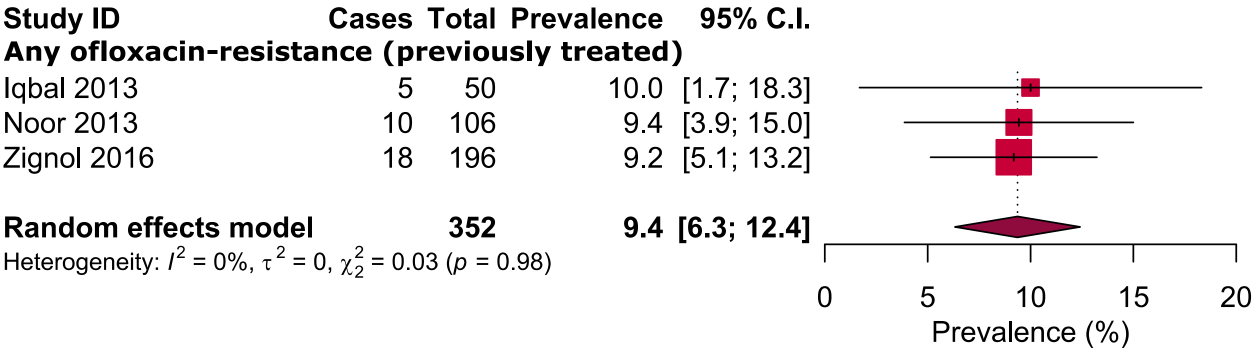
Study ID Cases Total Prevalence 95% C.I.
Any ofloxacin-resistance (previously treated)
Iqbal 2013 5 50 10.0 [1.7; 18.3]
Noor 2013 10 106 9.4 [3.9; 15.0]
Zignol 2016 18 196 9.2 [5.1; 13.2]
Random effects model 352 9.4 [6.3; 12.4]
Heterogeneity: I^2 = 0%, τ^2 = 0, χ^2_2 = 0.03 (p = 0.98)
0 5 10 15 20
Prevalence (%)

T

Study ID	Cases	Total	Prevalence	95% C.I.
**Any moxifloxacin-resistance (previously treated)**				
Heysell 2015	9	74	12.2	[4.7; 19.6]
Zignol 2016	3	192	1.6	[0.0; 3.3]
**Random effects model**		**266**	**6.2**	**[0.0; 16.5]**

Heterogeneity: I^2 = 86%, τ^2 = 0.0049, χ^2_1 = 7.37 (p < 0.01)

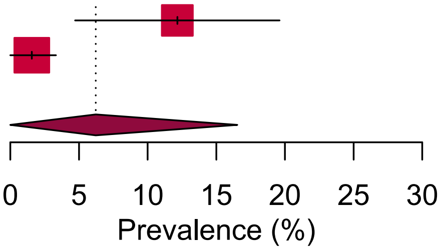

U

Study ID	Cases	Total	Prevalence	95% C.I.
**Any ethionamide-resistance (previously treated)**				
Heysell 2015	26	74	35.1	[24.3; 46]

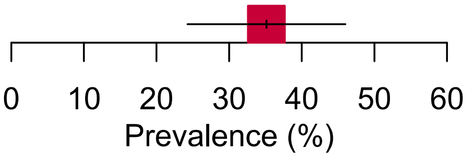

V

Study ID	Cases	Total	Prevalence	95% C.I.
**Any prothionamide-resistance (previously treated)**				
Aung 2014	83	477	17.4	[14.0; 20.8]
Van Deun 2010	60	427	14.1	[10.8; 17.3]
**Random effects model**		**904**	**15.7**	**[12.4; 19.0]**

Heterogeneity: I^2 = 48%, τ^2 = 0.0003, χ^2_1 = 1.92 (p = 0.17)

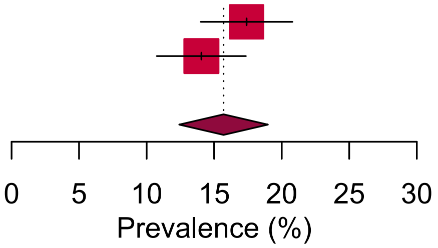

W

Study ID	Cases	Total	Prevalence	95% C.I.
**Any cycloserine-resistance (previously treated)**				
Heysell 2015	33	74	44.6	[33.3; 55.9]

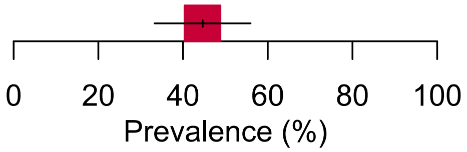

X

Study ID	Cases	Total	Prevalence	95% C.I.
**Any para-aminosalicylic acid-resistance (previously treated)**				
Heysell 2015	9	74	12.2	[4.7; 19.6]

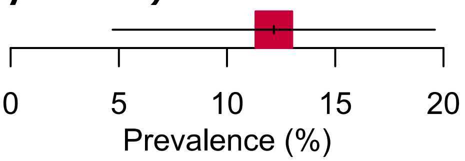

Y

Study ID	Cases	Total	Prevalence	95% C.I.
**Any levofloxacin-resistance (previously treated)**				
Zignol 2016	10	192	5.2	[2.1; 8.4]

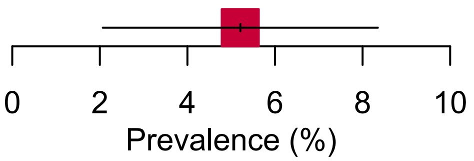

Figure S3: Any resistance to anti-TB drugs in newly diagnosed patients: A) streptomycin B) isoniazid, C) rifampicin, D) ethambutol E) pyrazinamide, F) gatifloxacin, G) kanamycin, H) levofloxacin, I) moxifloxacin, J) ofloxacin resistance and any resistance to anti-TB drugs in previously treated and newly diagnosed patients as well as any resistance to anti-TB drugs in previously treated patients: K) streptomycin, L) isoniazid, M) rifampicin, N) ethambutol, O) pyrazinamide, P) kanamycin, Q) amikacin, R) gatifloxacin, S) ofloxacin, T) moxifloxacin, U) ethionamide, V) prothionamide, W) cycloserine, X) para-aminosalicylic acid, and Y) levofloxacin.

A

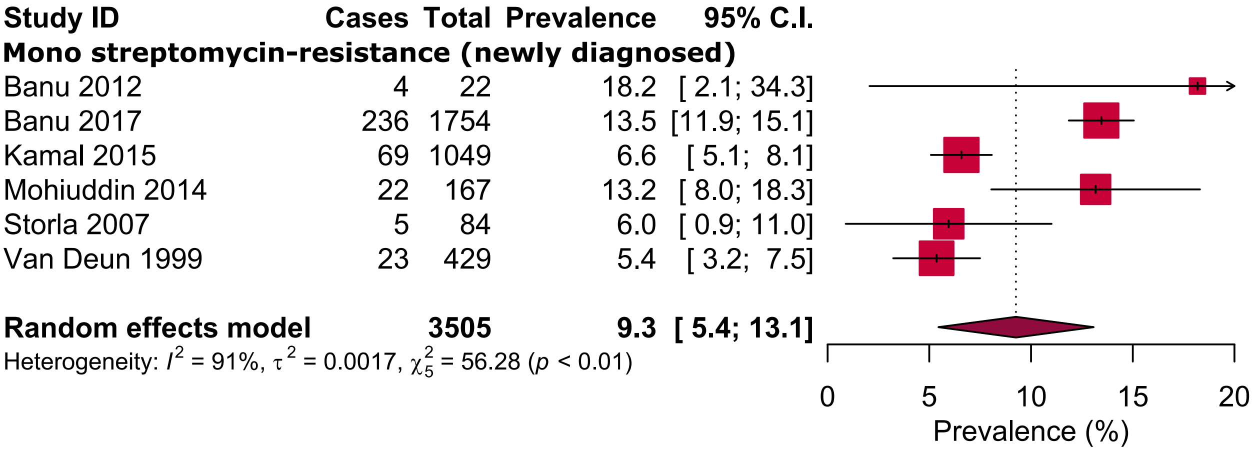

Study ID Cases Total Prevalence 95% C.I.
Mono streptomycin-resistance (newly diagnosed)
Banu 2012 4 22 18.2 [2.1; 34.3]
Banu 2017 236 1754 13.5 [11.9; 15.1]
Kamal 2015 69 1049 6.6 [5.1; 8.1]
Mohiuddin 2014 22 167 13.2 [8.0; 18.3]
Storla 2007 5 84 6.0 [0.9; 11.0]
Van Deun 1999 23 429 5.4 [3.2; 7.5]
Random effects model 3505 9.3 [5.4; 13.1]
Heterogeneity: I^2 = 91%, τ^2 = 0.0017, χ^2_5 = 56.28 (p < 0.01)
0 5 10 15 20
Prevalence (%)

B

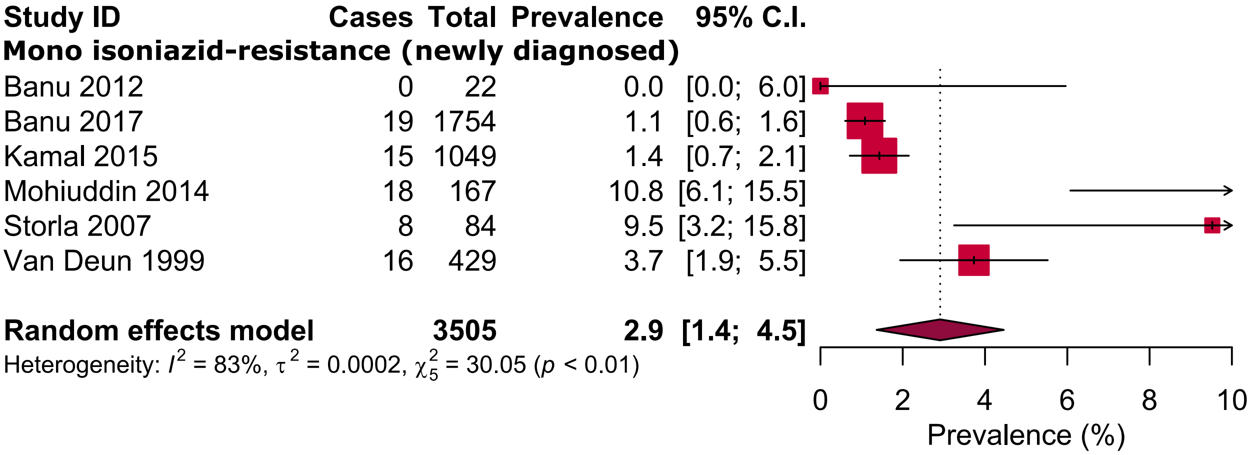

Study ID Cases Total Prevalence 95% C.I.
Mono isoniazid-resistance (newly diagnosed)
Banu 2012 0 22 0.0 [0.0; 6.0]
Banu 2017 19 1754 1.1 [0.6; 1.6]
Kamal 2015 15 1049 1.4 [0.7; 2.1]
Mohiuddin 2014 18 167 10.8 [6.1; 15.5]
Storla 2007 8 84 9.5 [3.2; 15.8]
Van Deun 1999 16 429 3.7 [1.9; 5.5]
Random effects model 3505 2.9 [1.4; 4.5]
Heterogeneity: I^2 = 83%, τ^2 = 0.0002, χ^2_5 = 30.05 (p < 0.01)
0 2 4 6 8 10
Prevalence (%)

C

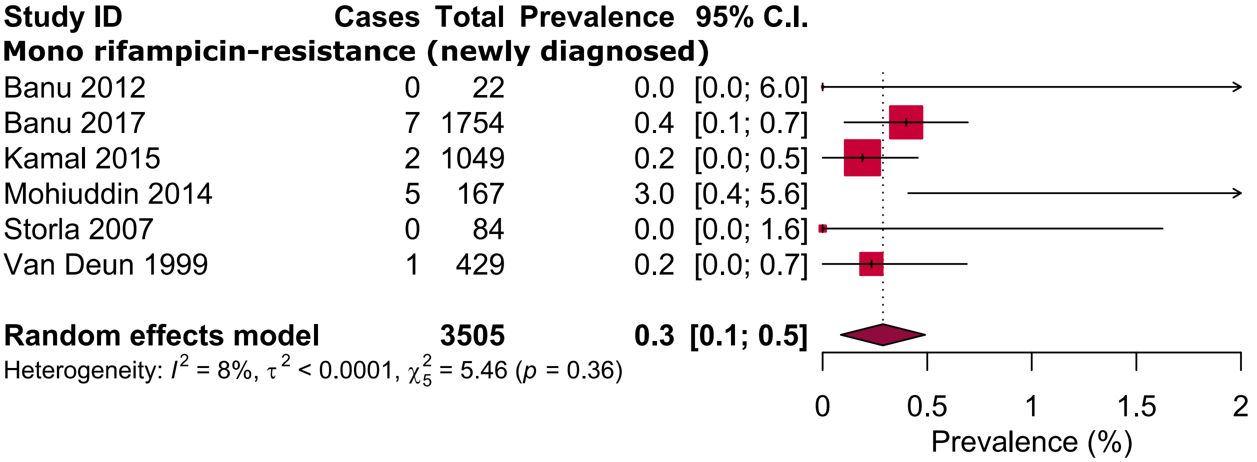

Study ID Cases Total Prevalence 95% C.I.
Mono rifampicin-resistance (newly diagnosed)
Banu 2012 0 22 0.0 [0.0; 6.0]
Banu 2017 7 1754 0.4 [0.1; 0.7]
Kamal 2015 2 1049 0.2 [0.0; 0.5]
Mohiuddin 2014 5 167 3.0 [0.4; 5.6]
Storla 2007 0 84 0.0 [0.0; 1.6]
Van Deun 1999 1 429 0.2 [0.0; 0.7]
Random effects model 3505 0.3 [0.1; 0.5]
Heterogeneity: I^2 = 8%, τ^2 < 0.0001, χ^2_5 = 5.46 (p = 0.36)
0 0.5 1 1.5 2
Prevalence (%)

D

Study ID	Cases	Total	Prevalence	95% C.I.
**Mono ethambutol-resistance (newly diagnosed)**				
Banu 2012	1	22	4.5	[0.0; 13.2]
Banu 2017	6	1754	0.3	[0.1; 0.6]
Kamal 2015	2	1049	0.2	[0.0; 0.5]
Mohiuddin 2014	8	167	4.8	[1.6; 8.0]
Storla 2007	1	84	1.2	[0.0; 3.5]
Van Deun 1999	0	429	0.0	[0.0; 0.3]
**Random effects model**		**3505**	**0.3**	**[0.0; 0.6]**

Heterogeneity: I^2 = 58%, τ^2 < 0.0001, χ^2_5 = 11.87 (p = 0.04)

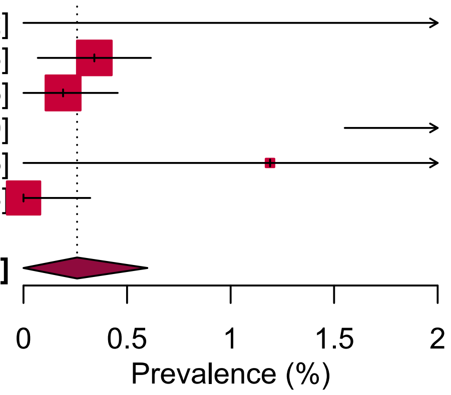

E

Study ID	Cases	Total	Prevalence	95% C.I.
**Mono streptomycin-resistance (previously treated)**				
Banu 2012	6	167	3.6	[0.8; 6.4]
Banu 2017	19	152	12.5	[7.2; 17.8]
Kamal 2015	21	291	7.2	[4.2; 10.2]
Mohiuddin 2014	2	25	8.0	[0.0; 18.6]
Storla 2007	1	11	9.1	[0.0; 26.1]
Van Deun 1999	10	216	4.6	[1.8; 7.4]
**Random effects model**		**862**	**6.5**	**[3.9; 9.1]**

Heterogeneity: I^2 = 52%, τ^2 = 0.0005, χ^2_5 = 10.44 (p = 0.06)

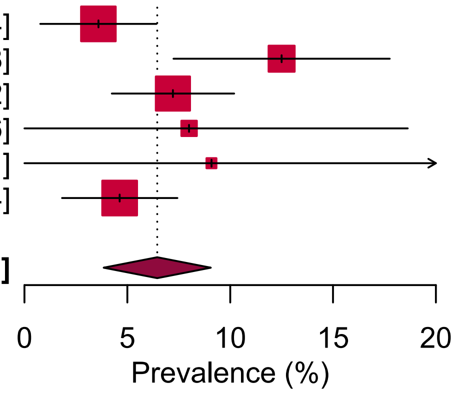

F

Study ID	Cases	Total	Prevalence	95% C.I.
**Mono isoniazid-resistance (previously treated)**				
Banu 2012	1	167	0.6	[0.0; 1.8]
Banu 2017	1	152	0.7	[0.0; 1.9]
Kamal 2015	7	291	2.4	[0.6; 4.2]
Mohiuddin 2014	0	25	0.0	[0.0; 5.3]
Noor 2013	9	109	8.3	[3.1; 13.4]
Storla 2007	1	11	9.1	[0.0; 26.1]
Van Deun 1999	28	216	13.0	[8.5; 17.4]
**Random effects model**		**971**	**3.5**	**[1.1; 5.9]**

Heterogeneity: I^2 = 84%, τ^2 = 0.0007, χ^2_6 = 37.73 (p < 0.01)

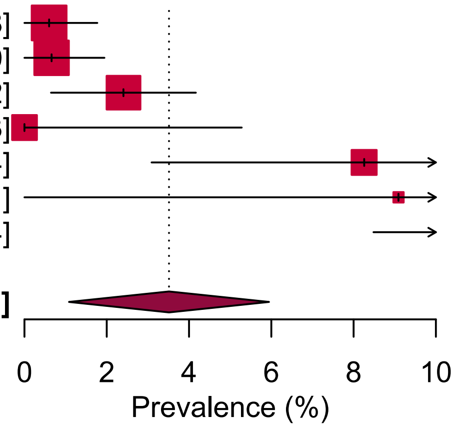

G

Study ID	Cases	Total	Prevalence	95% C.I.
**Mono rifampicin-resistance (previously treated)**				
Banu 2012	2	167	1.2	[0.0; 2.8]
Banu 2017	2	152	1.3	[0.0; 3.1]
Kamal 2015	1	291	0.3	[0.0; 1.0]
Mohiuddin 2014	0	25	0.0	[0.0; 5.3]
Noor 2013	2	109	1.8	[0.0; 4.4]
Storla 2007	0	11	0.0	[0.0; 11.3]
Van Deun 1999	2	216	0.9	[0.0; 2.2]
**Random effects model**		**971**	**0.7**	**[0.1; 1.2]**

Heterogeneity: $I^2 = 0\%$, $\tau^2 = 0$, $\chi^2_6 = 2.84$ ($p = 0.83$)

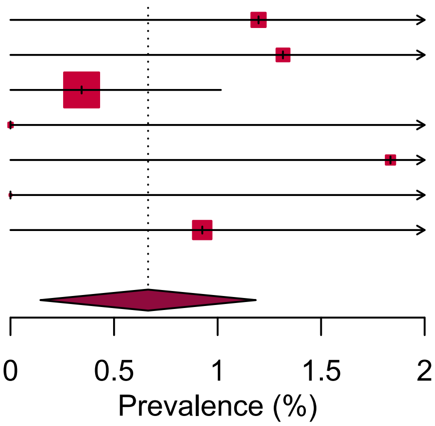

H

Study ID	Cases	Total	Prevalence	95% C.I.
**Mono ethambutol-resistance (previously treated)**				
Banu 2012	3	167	1.8	[0; 3.8]
Banu 2017	1	152	0.7	[0; 1.9]
Kamal 2015	0	291	0.0	[0; 0.5]
Mohiuddin 2014	2	25	8.0	[0; 18.6]
Storla 2007	0	11	0.0	[0; 11.3]
Van Deun 1999	3	216	1.4	[0; 2.9]
**Random effects model**		**862**	**0.7**	**[0; 1.5]**

Heterogeneity: $I^2 = 36\%$, $\tau^2 < 0.0001$, $\chi^2_5 = 7.80$ ($p = 0.17$)

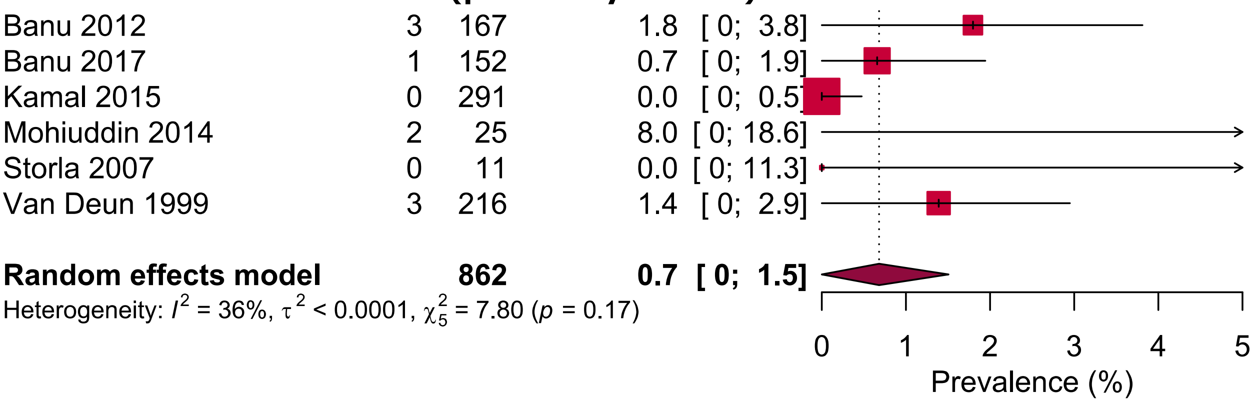

I

Study ID	Cases	Total	Prevalence	95% C.I.
**Mono ofloxacin-resistance (previously treated)**				
Noor 2013	0	109	0.0	[0; 1.3]

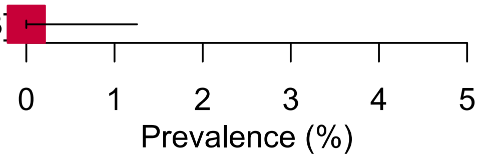

Figure S4: Mono resistance to anti-TB drugs in newly diagnosed patients: A) streptomycin, B) isoniazid, C) rifampicin, and D) ethambutol and mono resistance to anti- TB drugs in previously treated patients: E) streptomycin, F) isoniazid, G) rifampicin, H) ethambutol, and I) ofloxacin.

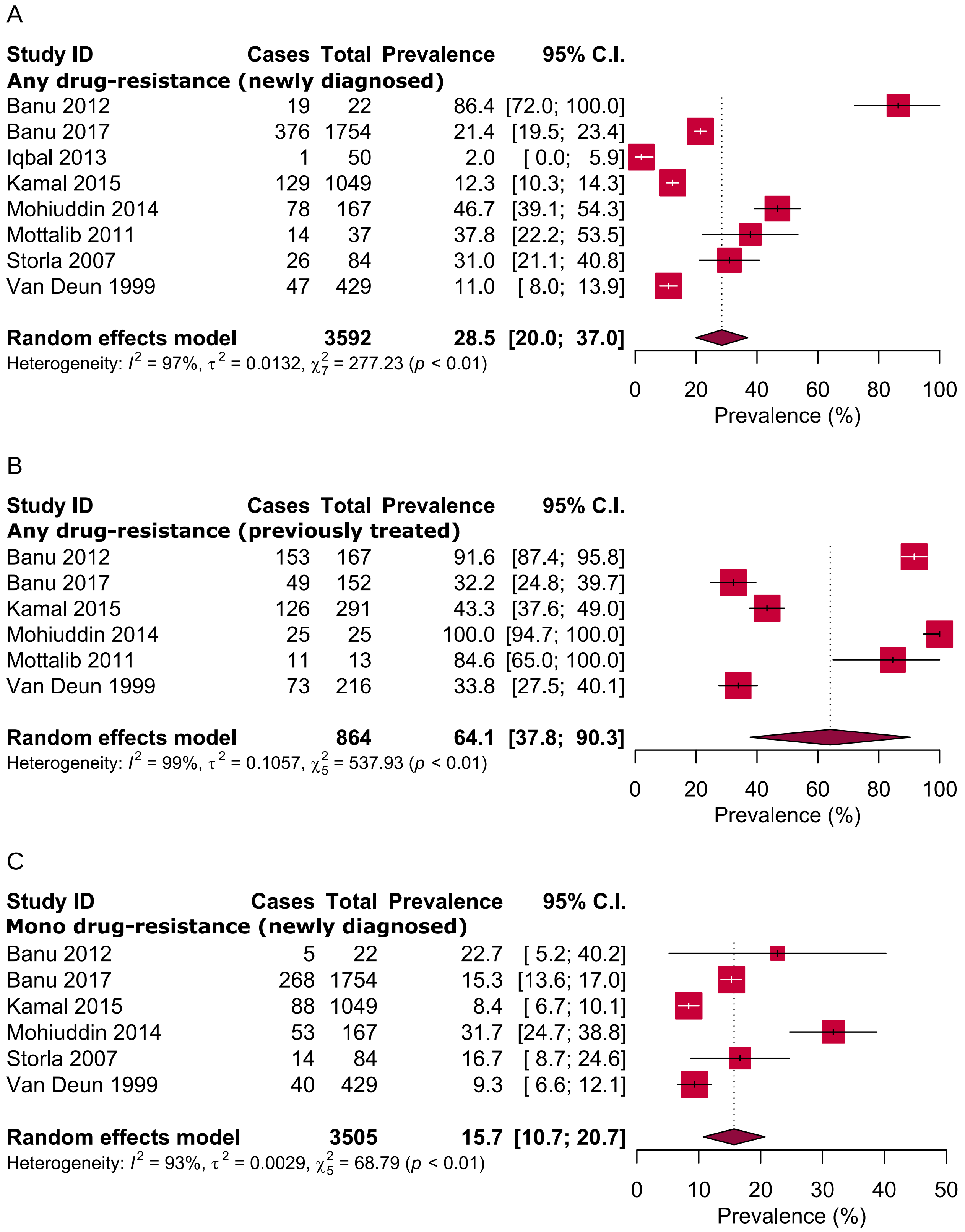
A
Study ID Cases Total Prevalence 95% C.I.
Any drug-resistance (newly diagnosed)
Banu 2012 19 22 86.4 [72.0; 100.0]
Banu 2017 376 1754 21.4 [19.5; 23.4]
Iqbal 2013 1 50 2.0 [0.0; 5.9]
Kamal 2015 129 1049 12.3 [10.3; 14.3]
Mohiuddin 2014 78 167 46.7 [39.1; 54.3]
Mottalib 2011 14 37 37.8 [22.2; 53.5]
Storla 2007 26 84 31.0 [21.1; 40.8]
Van Deun 1999 47 429 11.0 [8.0; 13.9]
Random effects model 3592 28.5 [20.0; 37.0]
Heterogeneity: I^2 = 97%, τ^2 = 0.0132, χ^2_7 = 277.23 (p < 0.01)
0 20 40 60 80 100
Prevalence (%)
B
Study ID Cases Total Prevalence 95% C.I.
Any drug-resistance (previously treated)
Banu 2012 153 167 91.6 [87.4; 95.8]
Banu 2017 49 152 32.2 [24.8; 39.7]
Kamal 2015 126 291 43.3 [37.6; 49.0]
Mohiuddin 2014 25 25 100.0 [94.7; 100.0]
Mottalib 2011 11 13 84.6 [65.0; 100.0]
Van Deun 1999 73 216 33.8 [27.5; 40.1]
Random effects model 864 64.1 [37.8; 90.3]
Heterogeneity: I^2 = 99%, τ^2 = 0.1057, χ^2_5 = 537.93 (p < 0.01)
0 20 40 60 80 100
Prevalence (%)
C
Study ID Cases Total Prevalence 95% C.I.
Mono drug-resistance (newly diagnosed)
Banu 2012 5 22 22.7 [5.2; 40.2]
Banu 2017 268 1754 15.3 [13.6; 17.0]
Kamal 2015 88 1049 8.4 [6.7; 10.1]
Mohiuddin 2014 53 167 31.7 [24.7; 38.8]
Storla 2007 14 84 16.7 [8.7; 24.6]
Van Deun 1999 40 429 9.3 [6.6; 12.1]
Random effects model 3505 15.7 [10.7; 20.7]
Heterogeneity: I^2 = 93%, τ^2 = 0.0029, χ^2_5 = 68.79 (p < 0.01)
0 10 20 30 40 50
Prevalence (%)

D

Study ID	Cases	Total	Prevalence	95% C.I.
**Mono drug-resistance (previously treated)**				
Banu 2012	12	167	7.2	[3.3; 11.1]
Banu 2017	23	152	15.1	[9.4; 20.8]
Kamal 2015	29	291	10.0	[6.5; 13.4]
Mohiuddin 2014	4	25	16.0	[1.6; 30.4]
Noor 2013	11	109	10.1	[4.4; 15.7]
Storla 2007	2	11	18.2	[0.0; 41.0]
Van Deun 1999	43	216	19.9	[14.6; 25.2]
**Random effects model**		**971**	**12.5**	**[8.7; 16.4]**

Heterogeneity: I^2 = 66%, τ^2 = 0.0015, χ^2_6 = 17.50 (p < 0.01)

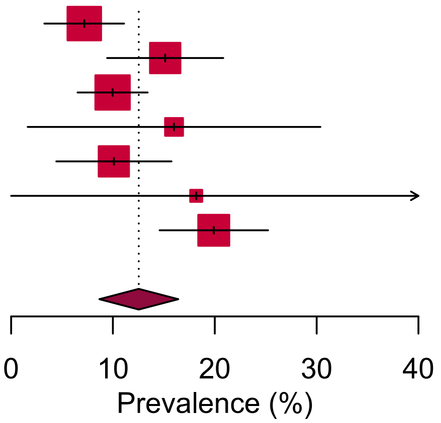

E

Study ID	Cases	Total	Prevalence	95% C.I.
**Multi drug-resistance (newly diagnosed)**				
Banu 2012	9	22	40.9	[20.4; 61.5]
Banu 2017	40	1754	2.3	[1.6; 3.0]
Hussain 2005	0	3908	0.0	[0.0; 0.0]
Kamal 2015	13	1049	1.2	[0.6; 1.9]
Mohiuddin 2014	7	167	4.2	[1.2; 7.2]
Storla 2007	2	84	2.4	[0.0; 5.6]
Van Deun 1999	1	429	0.2	[0.0; 0.7]
**Random effects model**		**7413**	**1.3**	**[0.3; 2.3]**

Heterogeneity: I^2 = 92%, τ^2 = 0.0001, χ^2_6 = 79.27 (p < 0.01)

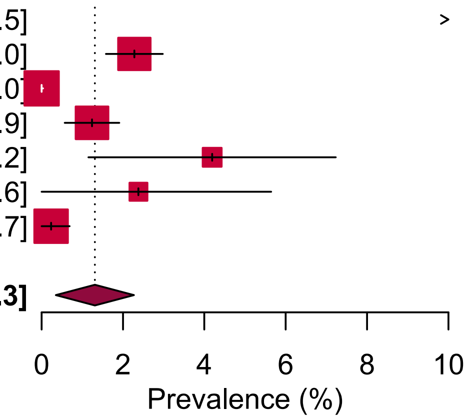

F

Study ID	Cases	Total	Prevalence	95% C.I.
**Multi drug-resistance (previously treated)**				
Banu 2012	15	167	9.0	[4.6; 13.3]
Banu 2017	21	152	13.8	[8.3; 19.3]
Hussain 2005	18	482	3.7	[2.0; 5.4]
Kamal 2015	83	291	28.5	[23.3; 33.7]
Mohiuddin 2014	9	25	36.0	[17.2; 54.8]
Noor 2013	82	109	75.2	[67.1; 83.3]
Storla 2007	0	11	0.0	[0.0; 11.3]
Van Deun 1999	12	216	5.6	[2.5; 8.6]
**Random effects model**		**1453**	**20.9**	**[9.6; 32.2]**

Heterogeneity: I^2 = 98%, τ^2 = 0.0248, χ^2_7 = 365.73 (p < 0.01)

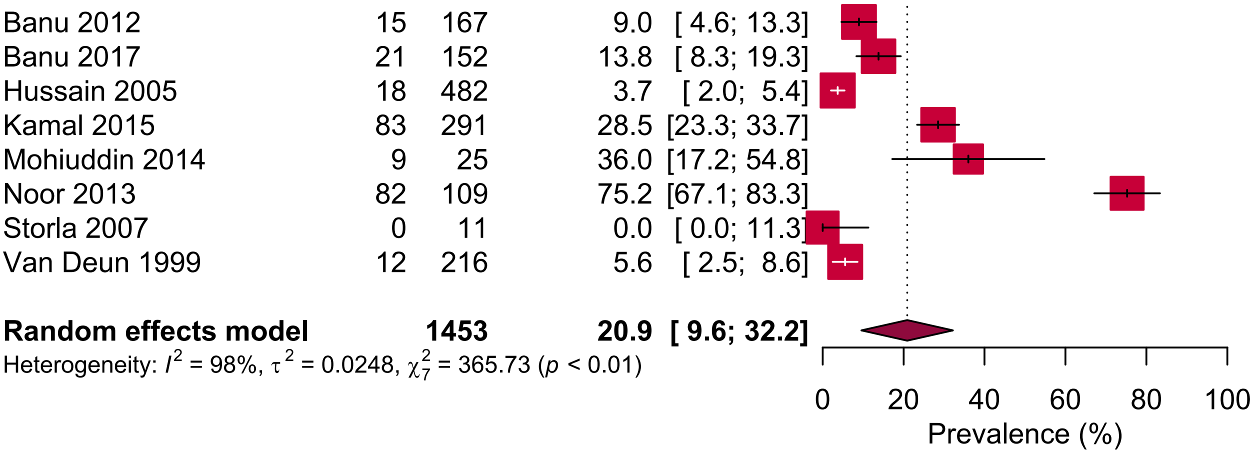

G

Study ID	Cases	Total	Prevalence	95% C.I.
**Poly drug-resistance (newly diagnosed)**				
Banu 2012	4	22	18.2	[2.1; 34.3]
Banu 2017	63	1754	3.6	[2.7; 4.5]
Kamal 2015	26	1049	2.5	[1.5; 3.4]
Mohiuddin 2014	18	167	10.8	[6.1; 15.5]
Storla 2007	9	84	10.7	[4.1; 17.3]
Van Deun 1999	6	429	1.4	[0.3; 2.5]
**Random effects model**		**3505**	**4.0**	**[2.2; 5.8]**

Heterogeneity: $I^2 = 83\%$, $\tau^2 = 0.0003$, $\chi^2_5 = 29.76$ ($p < 0.01$)

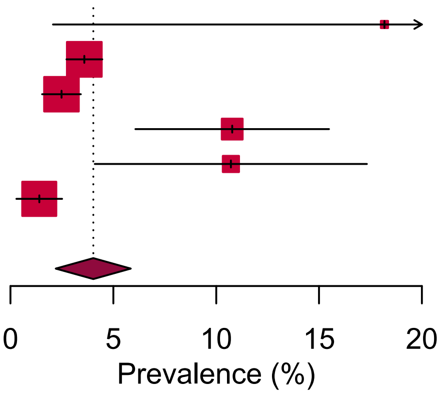

H

Study ID	Cases	Total	Prevalence	95% C.I.
**Poly drug-resistance (previously treated)**				
Banu 2012	12	167	7.2	[3.3; 11.1]
Banu 2017	10	152	6.6	[2.6; 10.5]
Kamal 2015	14	291	4.8	[2.4; 7.3]
Mohiuddin 2014	12	25	48.0	[28.4; 67.6]
Storla 2007	2	11	18.2	[0.0; 41.0]
Van Deun 1999	18	216	8.3	[4.6; 12.0]
**Random effects model**		**862**	**8.6**	**[4.5; 12.8]**

Heterogeneity: $I^2 = 76\%$, $\tau^2 = 0.0016$, $\chi^2_5 = 21.13$ ($p < 0.01$)

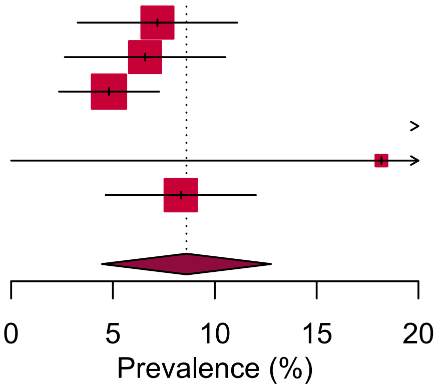

Figure S5: Overall DRs in newly diagnosed and previously treated TB patients. A) Any-DR in newly diagnosed patients, B) any-DR in previously treated patients, C) mono-DR in newly diagnosed patients, D) mono-DR in previously treated patients, E) multi-DR in newly diagnosed patients, F) multi-DR in previously treated patients, G) poly-DR in newly diagnosed patients, and H) poly-DR in previously treated patients.

A

Study ID	Cases	Total	Prevalence	95% C.I.
**Any drug-resistance (Excluding low-quality studies)**				
Afroz 2013	88	264	33.3	[27.6; 39.0]
Banu 2010	75	245	30.6	[24.8; 36.4]
Banu 2012	172	189	91.0	[86.9; 95.1]
Banu 2013	2	20	10.0	[0.0; 23.1]
Banu 2017	425	1906	22.3	[20.4; 24.2]
Kamal 2015	251	1340	18.7	[16.6; 20.8]
Khatun 2017	62	100	62.0	[52.5; 71.5]
Mohiuddin 2014	103	192	53.6	[46.6; 60.7]
Mottalib 2011	25	50	50.0	[36.1; 63.9]
Noor 2013	91	109	83.5	[76.5; 90.5]
Storla 2007	31	95	32.6	[23.2; 42.1]
Uddin 2018	108	244	44.3	[38.0; 50.5]
Van Deun 1999	120	645	18.6	[15.6; 21.6]
Wadud 2009	51	95	53.7	[43.7; 63.7]
Zaman 2005	318	657	48.4	[44.6; 52.2]
**Random effects model**		**6151**	**43.5**	**[31.7; 55.3]**

Heterogeneity: I^2 = 99%, τ^2 = 0.0528, χ^2_{14} = 1543.70 (p < 0.01)

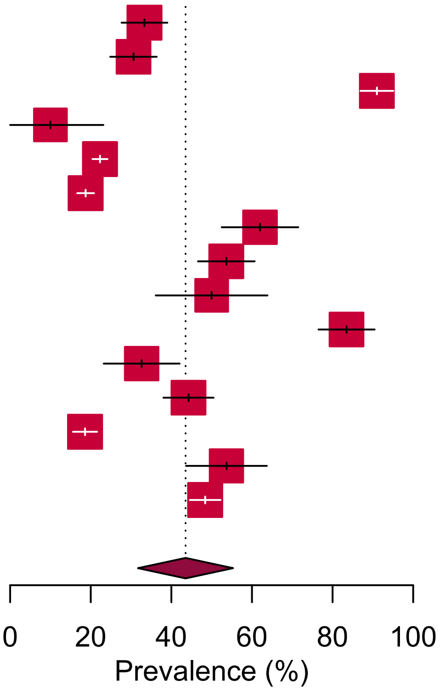

B

Study ID	Cases	Total	Prevalence	95% C.I.
**Multi drug-resistance (Excluding low-quality studies)**				
Afroz 2013	31	264	11.7	[7.9; 15.6]
Banu 2010	0	245	0.0	[0.0; 0.6]
Banu 2012	139	189	73.5	[67.3; 79.8]
Banu 2013	1	20	5.0	[0.0; 14.6]
Banu 2017	61	1906	3.2	[2.4; 4.0]
Kamal 2015	94	1340	7.0	[5.6; 8.4]
Khatun 2017	18	100	18.0	[10.5; 25.5]
Mohiuddin 2014	16	192	8.3	[4.4; 12.2]
Mottalib 2011	4	50	8.0	[0.5; 15.5]
Noor 2012	59	84	70.2	[60.5; 80.0]
Noor 2013	82	109	75.2	[67.1; 83.3]
Storla 2007	2	95	2.1	[0.0; 5.0]
Uddin 2018	39	244	16.0	[11.4; 20.6]
Van Deun 1999	13	645	2.0	[0.9; 3.1]
Wadud 2009	21	95	22.1	[13.8; 30.4]
Zaman 2005	36	657	5.5	[3.7; 7.2]
**Random effects model**		**6235**	**18.8**	**[14.7; 23.0]**

Heterogeneity: I^2 = 99%, τ^2 = 0.0064, χ^2_{15} = 1217.58 (p < 0.01)

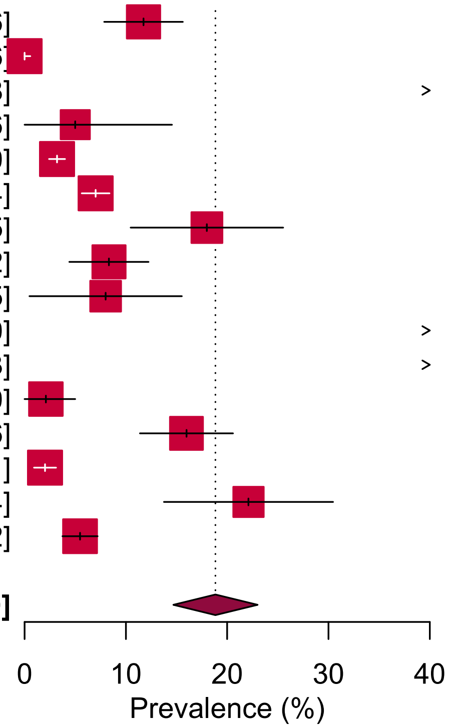

C

Study ID	Cases	Total	Prevalence	95% C.I.
**Extensive drug-resistance (Excluding low-quality studies)**				
Aung 2014	2	515	0.4	[0; 0.9]
Noor 2012	2	84	2.4	[0; 5.6]
Noor 2013	0	109	0.0	[0; 1.3]
Van deun 2010	0	427	0.0	[0; 0.3]
**Random effects model**		**1135**	**0.1**	**[0; 0.5]**

Heterogeneity: $I^2 = 11\%$, $\tau^2 < 0.0001$, $\chi^2_3 = 3.37$ ($p = 0.34$)

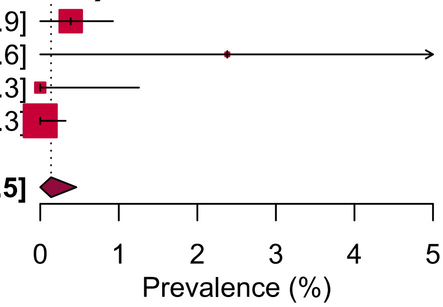

D

Study ID	Cases	Total	Prevalence	95% C.I.
**Any drug-resistance (Excluding small studies)**				
Afroz 2013	88	264	33.3	[27.6; 39.0]
Aurin 2014	198	277	71.5	[66.2; 76.8]
Banu 2010	75	245	30.6	[24.8; 36.4]
Banu 2012	172	189	91.0	[86.9; 95.1]
Banu 2017	425	1906	22.3	[20.4; 24.2]
Kamal 2015	251	1340	18.7	[16.6; 20.8]
Khatun 2017	62	100	62.0	[52.5; 71.5]
Mohiuddin 2014	103	192	53.6	[46.6; 60.7]
Noor 2013	91	109	83.5	[76.5; 90.5]
Uddin 2018	108	244	44.3	[38.0; 50.5]
Van Deun 1999	120	645	18.6	[15.6; 21.6]
Zaman 2005	318	657	48.4	[44.6; 52.2]
**Random effects model**		**6168**	**48.0**	**[34.2; 61.8]**

Heterogeneity: $I^2 = 99\%$, $\tau^2 = 0.0587$, $\chi^2_{11} = 1717.27$ ($p = 0$)

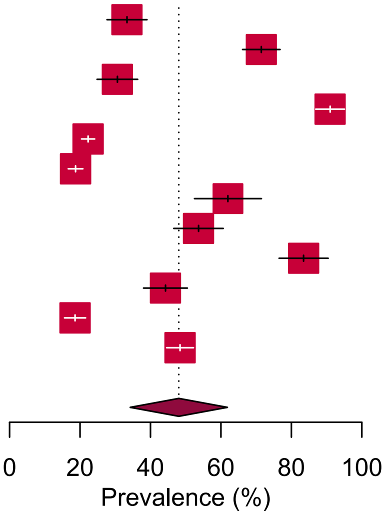

E

Study ID	Cases	Total	Prevalence	95% C.I.
**Mono drug-resistance (Excluding small studies)**				
Afroz 2013	31	264	11.7	[7.9; 15.6]
Banu 2010	51	245	20.8	[15.7; 25.9]
Banu 2012	12	189	6.3	[2.9; 9.8]
Banu 2017	291	1906	15.3	[13.7; 16.9]
Kamal 2015	117	1340	8.7	[7.2; 10.2]
Khatun 2017	19	100	19.0	[11.3; 26.7]
Mohiuddin 2014	57	192	29.7	[23.2; 36.2]
Noor 2013	11	109	10.1	[4.4; 15.7]
Uddin 2018	31	244	12.7	[8.5; 16.9]
Van Deun 1999	83	645	12.9	[10.3; 15.5]
**Random effects model**		**5234**	**14.1**	**[11.0; 17.3]**

Heterogeneity: $I^2 = 90\%$, $\tau^2 = 0.0021$, $\chi^2_9 = 88.03$ ($p < 0.01$)

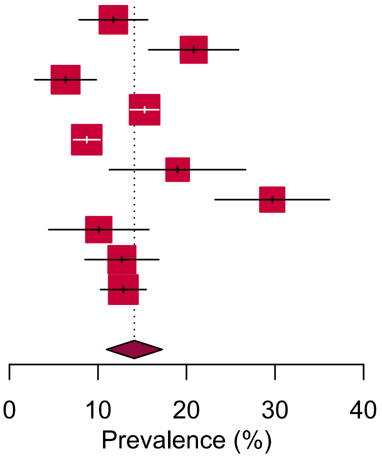

F

Study ID	Cases	Total	Prevalence	95% C.I.
**Multi drug-resistance (Excluding small studies)**				
Afroz 2013	31	264	11.7	[7.9; 15.6]
Aurin 2014	186	277	67.1	[61.6; 72.7]
Banu 2010	0	245	0.0	[0.0; 0.6]
Banu 2012	139	189	73.5	[67.3; 79.8]
Banu 2017	61	1906	3.2	[2.4; 4.0]
Hussain 2005	18	4390	0.4	[0.2; 0.6]
Kamal 2015	94	1340	7.0	[5.6; 8.4]
Khatun 2017	18	100	18.0	[10.5; 25.5]
Mohiuddin 2014	16	192	8.3	[4.4; 12.2]
Noor 2013	82	109	75.2	[67.1; 83.3]
Rahman 2009	221	363	60.9	[55.9; 65.9]
Uddin 2018	39	244	16.0	[11.4; 20.6]
Van Deun 1999	13	645	2.0	[0.9; 3.1]
Zaman 2005	36	657	5.5	[3.7; 7.2]
**Random effects model**		**10921**	**22.9**	**[19.0; 26.7]**

Heterogeneity: $I^2 = 99\%$, $\tau^2 = 0.0048$, $\chi^2_{13} = 2215.19$ ($p = 0$)

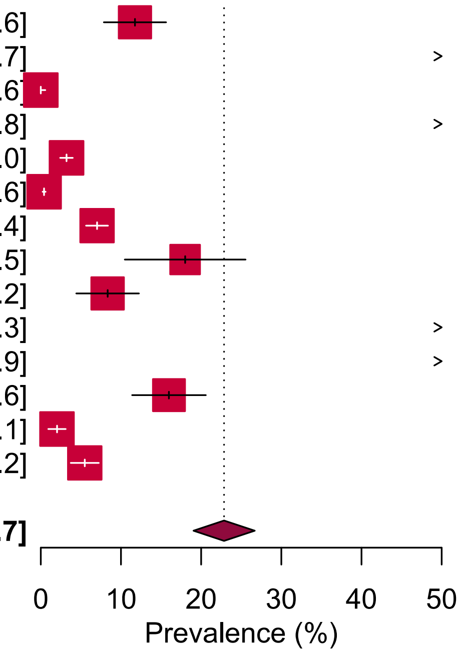

G

Study ID	Cases	Total	Prevalence	95% C.I.
**Poly drug-resistance (Excluding small studies)**				
Afroz 2013	26	264	9.8	[6.3; 13.4]
Banu 2010	24	245	9.8	[6.1; 13.5]
Banu 2012	16	189	8.5	[4.5; 12.4]
Banu 2017	73	1906	3.8	[3.0; 4.7]
Kamal 2015	40	1340	3.0	[2.1; 3.9]
Mohiuddin 2014	30	192	15.6	[10.5; 20.8]
Uddin 2018	38	244	15.6	[11.0; 20.1]
Van Deun 1999	24	645	3.7	[2.3; 5.2]
**Random effects model**		**5025**	**7.7**	**[5.5; 9.9]**

Heterogeneity: $I^2 = 90\%$, $\tau^2 = 0.0008$, $\chi^2_7 = 73.57$ ($p < 0.01$)

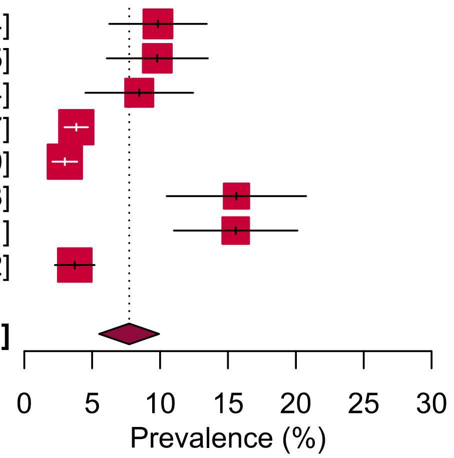

H

Study ID	Cases	Total	Prevalence	95% C.I.
**Extensive drug-resistance (Excluding small studies)**				
Aung 2014	2	515	0.4	[0; 0.9]
Noor 2013	0	109	0.0	[0; 1.3]
Van deun 2010	0	427	0.0	[0; 0.3]
**Random effects model**		**1051**	**0.1**	**[0; 0.4]**

Heterogeneity: $I^2 = 0\%$, $\tau^2 = 0$, $\chi^2_2 = 1.50$ ($p = 0.47$)

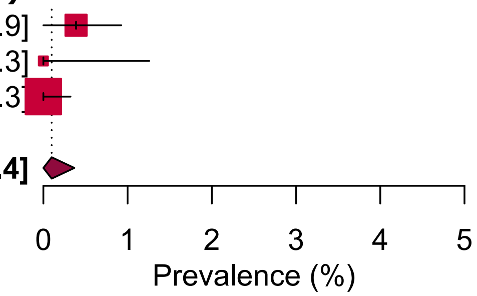

Figure S6: Sensitivity analyses: excluding low-quality studies from A) any drug-resistance, B) multi drug-resistance, C) extensive drug-resistance; excluding small studies from D) any drug-resistance, E) mono drug-resistance, F) multi drug-resistance, G) poly drug-resistance, and H) extensive drug-resistance.
